# Deep Learning for Cardiac Image Segmentation: A Review

**DOI:** 10.3389/fcvm.2020.00025

**Published:** 2020-03-05

**Authors:** Chen Chen, Chen Qin, Huaqi Qiu, Giacomo Tarroni, Jinming Duan, Wenjia Bai, Daniel Rueckert

**Affiliations:** ^1^Biomedical Image Analysis Group, Department of Computing, Imperial College London, London, United Kingdom; ^2^CitAI Research Centre, Department of Computer Science, City University of London, London, United Kingdom; ^3^School of Computer Science, University of Birmingham, Birmingham, United Kingdom; ^4^Data Science Institute, Imperial College London, London, United Kingdom; ^5^Department of Brain Sciences, Faculty of Medicine, Imperial College London, London, United Kingdom

**Keywords:** artificial intelligence, deep learning, neural networks, cardiac image segmentation, cardiac image analysis, MRI, CT, ultrasound

## Abstract

Deep learning has become the most widely used approach for cardiac image segmentation in recent years. In this paper, we provide a review of over 100 cardiac image segmentation papers using deep learning, which covers common imaging modalities including magnetic resonance imaging (MRI), computed tomography (CT), and ultrasound and major anatomical structures of interest (ventricles, atria, and vessels). In addition, a summary of publicly available cardiac image datasets and code repositories are included to provide a base for encouraging reproducible research. Finally, we discuss the challenges and limitations with current deep learning-based approaches (scarcity of labels, model generalizability across different domains, interpretability) and suggest potential directions for future research.

## 1. Introduction

Cardiovascular diseasess (CVDs) are the leading cause of death globally according to World Health Organization (WHO). About 17.9 million people died from CVDs in 2016, from CVD, mainly from heart disease and stroke^1^. The number is still increasing annually. In recent decades, major advances have been made in cardiovascular research and practice aiming to improve diagnosis and treatment of cardiac diseases as well as reducing the mortality of CVD. Modern medical imaging techniques, such as magnetic resonance imaging (MRI), computed tomography (CT) and ultrasound are now widely used, which enable non-invasive qualitative and quantitative assessment of cardiac anatomical structures and functions and provide support for diagnosis, disease monitoring, treatment planning, and prognosis.

**Figure 1:**
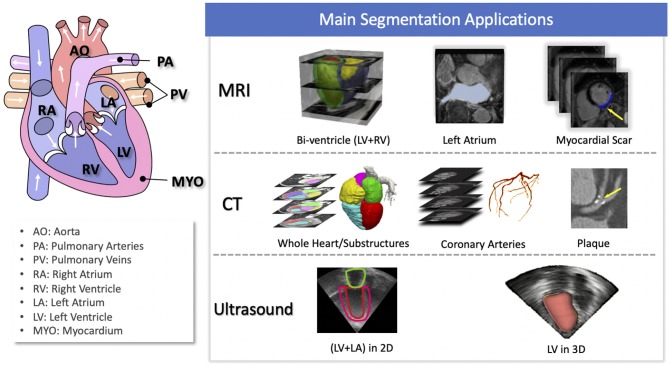
Overview of cardiac image segmentation tasks for different imaging modalities. For better understanding, we provide the anatomy of the heart on the left (image source: Wikimedia Commons, license: CC BY-SA 3.0). Of note, for simplicity, we list the tasks for which deep learning techniques have been applied, which will be discussed in section 3.

Of particular interest, cardiac image segmentation is an important first step in numerous applications. It partitions the image into a number of semantically (i.e., anatomically) meaningful regions, based on which quantitative measures can be extracted, such as the myocardial mass, wall thickness, left ventricle (LV) and right ventricle (RV) volume as well as ejection fraction (EF) etc. Typically, the anatomical structures of interest for cardiac image segmentation include the LV, RV, left atrium (LA), right atrium (RA), and coronary arteries. An overview of typical tasks related to cardiac image segmentation is presented in Figure 1, where applications for the three most commonly used modalities, i.e., MRI, CT, and ultrasound, are shown.

**Figure 2:**
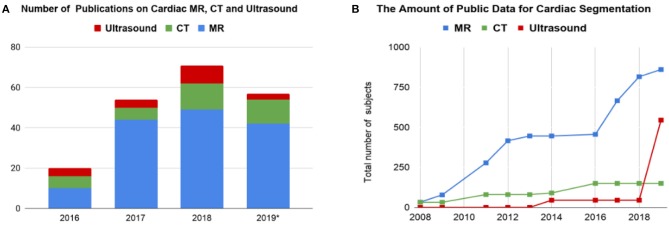
**(A)** Overview of numbers of papers published from 1st January 2016 to 1st August 2019 regarding deep learning-based methods for cardiac image segmentation reviewed in this work. **(B)** The increase of public data for cardiac image segmentation in the past 10 years. A list of publicly available datasets with detailed information is provided in Table 6. CT, computed tomography; MR, magnetic resonance.

Before the rise of deep learning, traditional machine learning techniques, such as model-based methods (e.g., active shape and appearance models) and atlas-based methods had been shown to achieve good performance in cardiac image segmentation (1–4). However, they often require significant feature engineering or prior knowledge to achieve satisfactory accuracy. In contrast, deep learning (DL)-based algorithms are good at *automatically* discovering intricate features from data for object detection and segmentation. These features are directly learned from data using a general-purpose learning procedure and in end-to-end fashion. This makes DL-based algorithms easy to apply to other image analysis applications. Benefiting from advanced computer hardware [e.g., graphical processing units (GPUs) and tensor processing units (TPUs)] as well as increased available data for training, DL-based segmentation algorithms have gradually outperformed previous state-of-the-art traditional methods, gaining more popularity in research. This trend can be observed in Figure 2A, which shows how the number of DL-based papers for cardiac image segmentation has increased strongly in the last years. In particular, the number of the publications for MR image segmentation is significantly higher than the numbers of the other two domains, especially in 2017. One reason, which can be observed in Figure 2B, is that the publicly available data for MR segmentation has increased remarkably since 2016.

In this paper, we provide an overview of state-of-the-art deep learning techniques for cardiac image segmentation in the three most commonly used modalities (i.e., MRI, CT, ultrasound) in clinical practice and discuss the advantages and remaining limitations of current deep learning-based segmentation methods that hinder widespread clinical deployment. To our knowledge, there have been several review papers that presented overviews about applications of DL-based methods for general medical image analysis (5–7), as well as some surveys dedicated to applications designed for cardiovascular image analysis (8, 9). However, none of them has provided a systematic overview focused on *cardiac segmentation applications*. This review paper aims at providing a comprehensive overview from the debut to the state-of-the-art of deep learning algorithms, focusing on a variety of cardiac image segmentation tasks (e.g., the LV, RV, and vessel segmentation) (section 3). Particularly, we aim to cover most influential DL-related works in this field published until 1st August 2019 and categorized these publications in terms of specific methodology. Besides, in addition to the basics of deep learning introduced in section 2, we also provide a summary of public datasets (see **Table 6**) as well as public code (see **Table 7**), aiming to present a good reading basis for newcomers to the topic and encourage future contributions. More importantly, we provide insightful discussions about the current research situations (section 3.4) as well as challenges and potential directions for future work (section 4).

### 1.1. Search Criterion

To identify related contributions, search engines like Scopus and PubMed were queried for papers containing (“convolutional” OR “deep learning”) and (“cardiac”) and (“image segmentation”) in title or abstract. Additionally, conference proceedings for MICCAI, ISBI, and EMBC were searched based on the titles of papers. Papers which do not primarily focus on segmentation problems were excluded. The last update to the included papers was on Aug 1, 2019.

## 2. Fundamentals of Deep Learning

Deep learning models are deep artificial neural networks. Each neural network consists of an input layer, an output layer, and multiple hidden layers. In the following section, we will review several deep learning networks and key techniques that have been commonly used in state-of-the-art segmentation algorithms. For a more detailed and thorough illustration of the mathematical background and fundamentals of deep learning we refer the interested reader to Goodfellow (43).

### 2.1. Neural Networks

In this section, we first introduce basic neural network architectures and then briefly introduce building blocks which are commonly used to boost the ability of the networks to learn features that are useful for image segmentation.

#### 2.1.1. Convolutional Neural Networks (CNNs)

In this part, we will introduce convolutional neural network (CNN), which is the most common type of deep neural networks for image analysis. CNN have been successfully applied to advance the state-of-the-art on many image classification, object detection and segmentation tasks.

**Figure 3:**
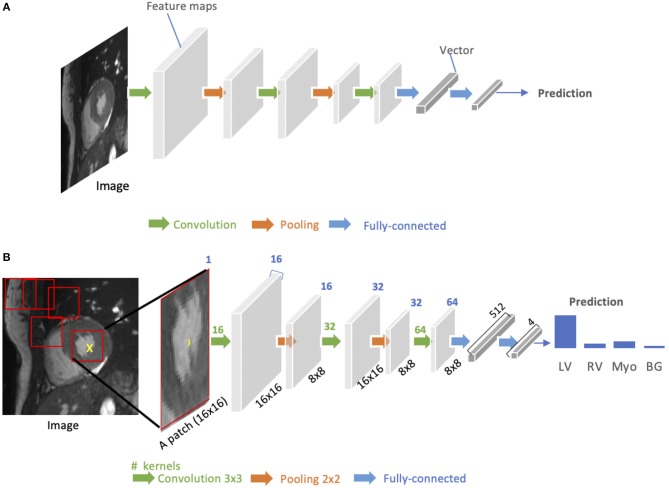
**(A)** Generic architecture of convolutional neural networks (CNN). A CNN takes a cardiac MR image as input, learning hierarchical features through a stack of convolutions and pooling operations. These spatial feature maps are then flattened and reduced into a vector through fully connected layers. This vector can be in many forms, depending on the specific task. It can be probabilities for a set of classes (image classification) or coordinates of a bounding box (object localization) or a predicted label for the center pixel of the input (patch-based segmentation) or a real value for regression tasks (e.g., left ventricular volume estimation). **(B)** Patch-based segmentation method based on a CNN classifier. The CNN takes a patch as input and outputs the probabilities for four classes where the class with the highest score is the prediction for the center pixel (see the yellow cross) in this patch. By repeatedly forwarding patches located at different locations into the CNN for classification, one can finally get a pixel-wise segmentation map for the whole image. LV, left ventricle cavity; RV, right ventricle cavity; BG, Background; Myo, left ventricular myocardium. The blue number at the top indicates the number of channels of the feature maps. Here, each convolution kernel is a 3 × 3 kernel (stride = 1, padding = 1), which will produces an output feature map with the same height and width as the input.

As shown in Figure 3A, a standard CNN consists of an input layer, an output layer and a stack of functional layers in between that transform an input into an output in a specific form (e.g., vectors). These functional layers often contains convolutional layers, pooling layers and/or fully-connected layers. In general, a convolutional layer CONV_*l*_ contains *k*_*l*_ convolution kernels/filters, which is followed by a normalization layer [e.g., batch normalization (44)] and a non-linear activation function [e.g., rectified linear unit (ReLU)] to extract *k*_*l*_ feature maps from the input. These feature maps are then downsampled by pooling layers, typically by a factor of 2, which remove redundant features to improve the statistical efficiency and model generalization. After that, fully connected layers are applied to reduce the dimension of features from its previous layer and find the most task-relevant features for inference. The output of the network is a fix-sized vector where each element can be a probabilistic score for each category (for image classification), a real value for a regression task (e.g., the left ventricular volume estimation) or a set of values (e.g., the coordinates of a bounding box for object detection and localization).

A key component of CNN is the convolutional layer. Each convolutional layer has *k*_*l*_ convolution kernels to extract *k*_*l*_ feature maps and the size of each kernel *n* is chosen to be small in general, e.g., *n* = 3 for a 2D 3 × 3 kernel, to reduce the number of parameters^2^. While the kernels are small, one can increase the receptive field (the area of the input image that potentially impacts the activation of a particular convolutional kernel/neuron) by increasing the number of convolutional layers. For example, a convolutional layer with large 7 × 7 kernels can be replaced by three layers with small 3 × 3 kernels (45). The number of weights is reduced by a factor of 7^2^/(3 × (3^2^)) ≈ 2 while the receptive field remains the same (7 × 7). An online resource^3^ is referred here, which illustrates and visualizes the change of receptive field by varying the number of hidden layers and the size of kernels. In general, increasing the depth of convolution neural networks (the number of hidden layers) to enlarge the receptive field can lead to improved model performance, e.g., classification accuracy (45).

CNNs for image classification can also be employed for image segmentation applications without major adaptations to the network architecture (46), as shown in Figure 3B. However, this requires to divide each image into patches and then train a CNN to predict the class label of the center pixel for every patch. One major disadvantage of this patch-based approach is that, at inference time, the network has to be deployed for every patch individually despite the fact that there is a lot of redundancy due to multiple overlapping patches in the image. As a result of this inefficiency, the main application of CNNs with fully connected layers for cardiac segmentation is object localization, which aims to estimate the bounding box of the object of interest in an image. This bounding box is then used to crop the image, forming an image pre-processing step to reduce the computational cost for segmentation (47). For efficient, end-to-end pixel-wise segmentation, a variant of CNNs called fully convolutional neural network (FCN) is more commonly used, which will be discussed in the next section.

#### 2.1.2. Fully Convolutional Neural Networks (FCNs)

**Figure 4:**
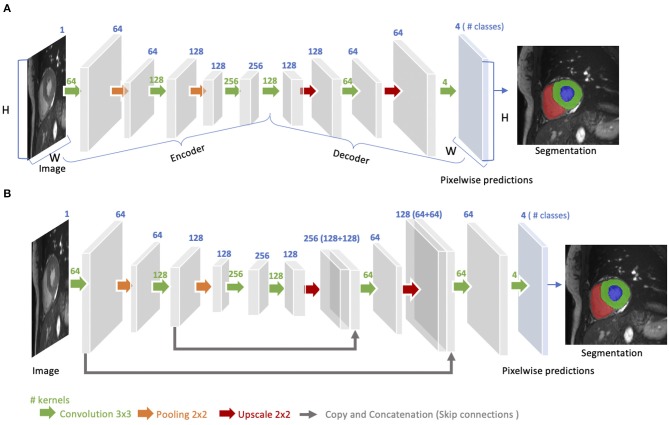
**(A)** Architecture of a fully convolutional neural network (FCN). The FCN first takes the whole image as input, learns image features though the encoder, gradually recovers the spatial dimension by a series of upscaling layers (e.g., transposed convolution layers, unpooling layers) in the decoder and then produce 4-class pixel-wise probabilistic maps to predict regions of the left ventricle cavity (blue region), the left ventricular myocardium (green region) and the right ventricle cavity (red region) and background. The final segmentation map is obtained by assigning each pixel with the class of the highest probability. One use case of this FCN-based cardiac segmentation can be found in Tran (24). **(B)** Architecture of a U-net. On the basis of FCN, U-net adds “skip connections” (gray arrows) to aggregate feature maps from coarse to fine through concatenation and convolution operations. For simplicity, we reduce the number of downsampling and upsampling blocks in the diagram. For detailed information, we recommend readers to the original paper (49).

The idea of FCN was first introduced by Long et al. (48) for image segmentation. FCNs are a special type of CNNs that do not have any fully connected layers. In general, as shown in Figure 4A, FCNs are designed to have an encoder-decoder structure such that they can take input of arbitrary size and produce the output with the same size. Given an input image, the encoder first transforms the input into high-level feature representation whereas the decoder interprets the feature maps and recovers spatial details back to the image space for pixel-wise prediction through a series of upsampling and convolution operations. Here, upsampling can be achieved by applying transposed convolutions, e.g., 3 × 3 transposed convolutional kernels with a stride of 2 to up-scale feature maps by a factor of 2. These transposed convolutions can also be replaced by unpooling layers and upsampling layers. Compared to a patch-based CNN for segmentation, FCN is trained and applied to the entire images, removing the need for patch selection (50).

FCN with the simple encoder-decoder structure in Figure 4A may be limited to capture detailed context information in an image for precise segmentation as some features may be eliminated by the pooling layers in the encoder. Several variants of FCNs have been proposed to propagate features from the encoder to the decoder, in order to boost the segmentation accuracy. The most well-known and most popular variant of FCNs for biomedical image segmentation is the U-net (49). On the basis of the vanilla FCN (48), the U-net employs skip connections between the encoder and decoder to recover spatial context loss in the down-sampling path, yielding more precise segmentation (see Figure 4B). Several state-of-the-art cardiac image segmentation methods have adopted the U-net or its 3D variants, the 3D U-net (51) and the 3D V-net (52), as their backbone networks, achieving promising segmentation accuracy for a number of cardiac segmentation tasks (26, 53, 54).

#### 2.1.3. Recurrent Neural Networks (RNNs)

**Figure 5:**
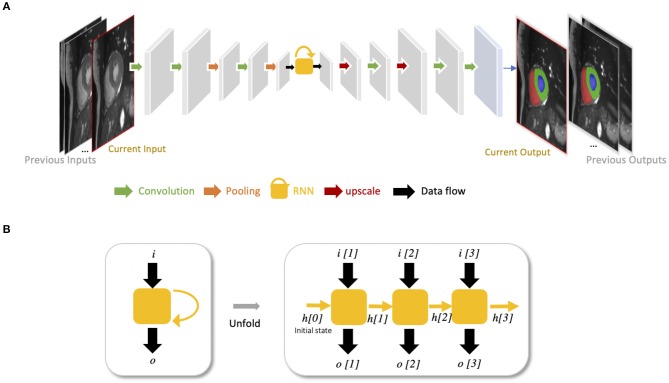
**(A)** Example of FCN with an RNN for cardiac image segmentation. The yellow block with a curved arrow represents a RNN module, which utilizes the knowledge learned from the past to make the current decision. In this example, the network is used to segment cardiac ventricles from a stack of 2D cardiac MR slices, which allows propagation of contextual information from adjacent slices for better inter-slice coherence (55). This type of RNN is also suitable for sequential data, such as cine MR images and ultrasound movies to learn temporal coherence. **(B)** Unfolded schema of the RNN module for visualizing the inner process when the input is a sequence of three images. Each time, this RNN module will receive an input *i*[*t*] at time step *t*, and produce an output *o*[*t*], considering not only the input information but also the hidden state (“memory”) *h*[*t* − 1] from the previous time step *t*−1.

Recurrent neural networks (RNNs) are another type of artificial neural networks which are used for sequential data, such as cine MRI and ultrasound image sequences. An RNN can “remember” the past and use the knowledge learned from the past to make its present decision (see Figures 5A,B). For example, given a sequence of images, an RNN takes the first image as input, captures the information to make a prediction and then memorize this information which is then utilized to make a prediction for the next image. The two most widely used architectures in the family of RNNs are LSTM (56) and gated recurrent unit (GRU) (57), which are capable of modeling long-term memory. A use case for cardiac segmentation is to combine an RNN with a 2D FCN so that the combined network is capable of capturing information from adjacent slices to improve the inter-slice coherence of segmentation results (55).

#### 2.1.4. Autoencoders (AE)

**Figure 6:**
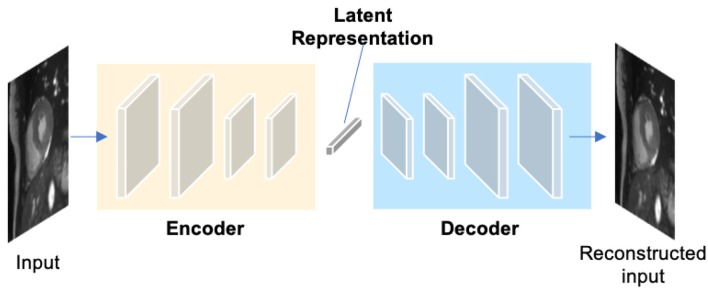
A generic architecture of an autoencoder. An autoencoder employs an encoder-decoder structure, where the encoder maps the input data to a low-dimensional latent representation and the decoder interprets the code and reconstructs the input. The learned latent representation has been found effective for cardiac image segmentation (58, 59), cardiac shape modeling (60) and cardiac segmentation correction (61).

Autoencoders (AEs) are a type of neural networks that are designed to learn compact latent representations from data without supervision. A typical architecture of an autoencoder consists of two networks: an encoder network and a decoder network for the reconstruction of the input (see Figure 6). Since the learned representations contain generally useful information in the original data, many researchers have employed autoencoders to extract general semantic features or shape information from input images or labels and then use those features to guide the cardiac image segmentation (58, 62, 63).

#### 2.1.5. Generative Adversarial Networks (GAN)

**Figure 7:**
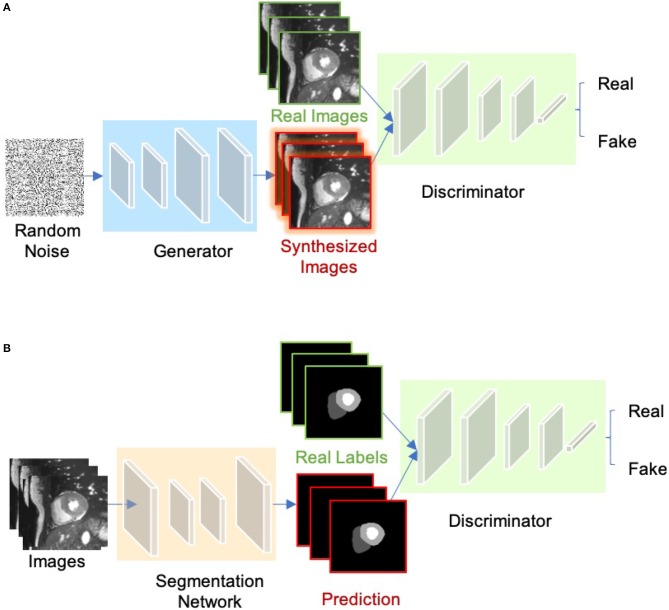
**(A)** Overview of GAN for image synthesis. **(B)** Overview of adversarial training for image segmentation.

The concept of Generative adversarial network (GAN) was proposed by Goodfellow et al. (64) for image synthesis from noise. GANs are a type of generative models that learn to model the data distribution of real data and thus are able to create new image examples. As shown in Figure 7A, a GAN consists of two networks: a generator network and a discriminator network. During training, the two networks are trained to compete against each other: the generator produces fake images aimed at fooling the discriminator, whereas the discriminator tries to identify real images from fake ones. This type of training is referred to as “adversarial training,” since the two models are both set to win the competition. This training scheme can also be used for training a segmentation network. As shown in Figure 7B, the generator is replaced by a segmentation network and the discriminator is required to distinguish the generated segmentation maps from the ground truth ones (the target segmentation maps). In this way, the segmentation network is encouraged to produce more anatomically plausible segmentation maps (65, 66).

#### 2.1.6. Advanced Building Blocks for Improved Segmentation

**Figure 8:**
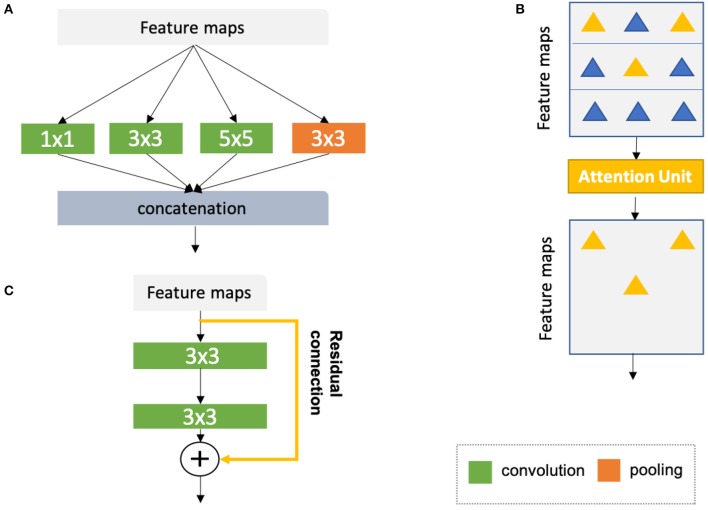
**(A)** Naive version of the inception module (44). In this module, convolutional kernels with varying sizes are applied to the same input for multi-scale feature fusion. On the basis of the naive structure, a family of advanced inception modules with more complex structures have been developed (67, 68). **(B)** Schematic diagram of the attention module (69, 70). The attention module teaches the network to pay attention to important features (e.g., features relevant to anatomy) and ignore redundant features. **(C)** Schematic diagram of a residual unit (71). The yellow arrow represents a residual connection which is applied to reusing the features from a previous layer. The numbers in the green and orange blocks denote the sizes of corresponding convolutional or pooling kernels. Here, for simplicity, all diagrams have been reproduced based on the illustration in the original papers.

Medical image segmentation, as an important step for quantitative analysis and clinical research, requires high pixel-wise accuracy. Over the past years, many researchers have developed advanced building blocks to learn robust, representative features for precise segmentation. These techniques have been widely applied to state-of-the-art neural networks (e.g., U-net) to improve cardiac image segmentation performance. Therefore, we identified several important techniques reported in the literature to this end and present them with corresponding references for further reading. These techniques are:

1. Advanced convolutional modules for multi-scale feature aggregation:
  - Inception modules (44, 67, 68), which concatenate multiple convolutional filter banks with different kernel sizes to extract multi-scale features in parallel (see Figure 8A);
  - Dilated convolutional kernels (72), which are modified convolution kernels with the same kernel size but different kernel strides to process input feature maps at larger scales;
  - Deep supervision (73), which utilizes the outputs from multiple intermediate hidden layers for multi-scale prediction;
  - Atrous spatial pyramid pooling (74), which applies spatial pyramid pooling (75) with various kernel strides to input feature maps for multi-scale feature fusion;
2. Adaptive convolutional kernels designed to focus on important features:
  - Attention units (69, 70, 76), which learn to adaptively recalibrate features spatially (see Figure 8B);
  - Squeeze-and-excitation blocks (77), which are used to recalibrate features with learnable weights across channels;
3. Interlayer connections designed to reuse features from previous layers:
  - Residual connections (71), which add outputs from a previous layer to the feature maps learned from the current layer (see Figure 8C);
  - Dense connections (78), which concatenate outputs from all preceding layers to the feature maps learned from the current layer.

### 2.2. Training Neural Networks

Before being able to perform inference, neural networks must be trained. Standard training process requires a dataset that contains paired images and labels {***x***, ***y***} for training and testing, an optimizer (e.g., stochastic gradient descent, Adam) and a loss function to update the model parameters. This function accounts for the error of the network prediction in each iteration during training, providing signals for the optimizer to update the network parameters through backpropagation (43, 79). The goal of training is to find proper values of the network parameters to minimize the loss function.

#### 2.2.1. Common Loss Functions

For regression tasks (e.g., heart localization, calcium scoring, landmark detection, image reconstruction), the simplest loss function is the mean squared error (MSE):

(1) $$\begin{array}{l} L_{\text{MSE}}=\frac{1}{n}\overset{n}{\underset{i=1}{\sum }}{\left(y_{i}-{\hat{y}}_{i}\right)}^{2}, \end{array}$$

where ***y***_*i*_ is the vector of target values and ${\hat{y}}_{i}$ is the vector of the predicted values; *n* is the number of data samples at each iteration.

Cross-entropy is the most common loss for both image classification and segmentation tasks. In particular, the cross-entropy loss for segmentation summarizes pixel-wise probability errors between a predicted probabilistic output $p_{i}^{c}$ and its corresponding target segmentation map $y_{i}^{c}$ for each class *c*^4^:

(2) $$\begin{array}{l} L_{\text{CE}}=-\frac{1}{n}\overset{n}{\underset{i=1}{\sum }}\overset{C}{\underset{c=1}{\sum }}y_{i}^{c}\log\left(p_{i}^{c}\right), \end{array}$$

where *C* is the number of all classes. Another loss function which is specifically designed for object segmentation is called soft-Dice loss function (52), which penalizes the mismatch between a predicted segmentation map and its target map at pixel-level:

(3) $$\begin{array}{l} L_{Dice}=1-\frac{2\sum_{i=1}^{n}\sum_{c=1}^{C}y_{i}^{c}p_{i}^{c}}{\sum_{i=1}^{n}\sum_{c=1}^{C}\left(y_{i}^{c}+p_{i}^{c}\right)}. \end{array}$$

In addition, there are several variants of the cross-entropy or soft-Dice loss, such as the weighted cross-entropy loss (25, 80) and weighted soft-Dice loss (29, 81) that are used to address potential class imbalance problem in medical image segmentation tasks where the loss term is weighted to account for rare classes or small objects.

#### 2.2.2. Reducing Over-Fitting

The biggest challenge of training deep networks for medical image analysis is over-fitting, due to the fact that there is often a limited number of training images in comparison with the number of learnable parameters in a deep network. A number of techniques have been developed to alleviate this problem. Some of the techniques are the following ones:

- **Weight regularization**: Weight regularization is a type of regularization techniques that add weight penalties to the loss function. Weight regularization encourages small or zero weights for less relevant or irrelevant inputs. Common methods to constrain the weights include L1 and L2 regularization, which penalize the sum of the absolute weights and the sum of the squared weights, respectively;
- **Dropout** (82): Dropout is a regularization method that randomly drops some units from the neural network during training, encouraging the network to learn a sparse representation;
- **Ensemble learning**: Ensemble learning is a type of machine learning algorithms that combine multiple trained models to obtain better predictive performance than individual models, which has been shown effective for medical image segmentation (83, 84);
- **Data augmentation**: Data augmentation is a training strategy that artificially generates more training samples to increase the diversity of the training data. This can be done via applying affine transformations (e.g., rotation, scaling), flipping or cropping to original labeled samples;
- **Transfer learning**: Transfer learning aims to transfer knowledge from one task to another related but different target task. This is often achieved by reusing the weights of a pre-trained model, to initialize the weights in a new model for the target task. Transfer learning can help to decrease the training time and achieve lower generalization error (85).

### 2.3. Evaluation Metrics

To quantitatively evaluate the performance of automated segmentation algorithms, three types of metrics are commonly used: (a) volume-based metrics (e.g., Dice metric, Jaccard similarity index); (b) surface distance-based metrics (e.g., mean contour distance, Hausdorff distance); (c) clinical performance metrics (e.g., ventricular volume and mass). For a detailed illustration of common used clinical indices in cardiac image analysis, we recommend the review paper by Peng et al. (2). In our paper, we mainly report the accuracy of methods in terms of the Dice metric for ease of comparison. The Dice score measures the ratio of overlap between two results (e.g., automatic segmentation vs. manual segmentation), ranging from 0 (mismatch) to 1 (perfect match). It is also important to note that the segmentation accuracy of different methods are not directly comparable in general, unless these methods are evaluated on the same dataset. This is because, even for the same segmentation task, different datasets can have different imaging modalities, different patient populations and different methods of image acquisition, which will affect the task complexities and result in different segmentation performances.

## 3. Deep Learning for Cardiac Image Segmentation

In this section, we provide a summary of deep learning-based applications for the three main imaging modalities: MRI, CT, and ultrasound regarding specific applications for targeted structures. In general, these deep learning-based methods provide an efficient and effective way to segmenting particular organs or tissues (e.g., the LV, coronary vessels, scars) in different modalities, facilitating follow-up quantitative analysis of cardiovascular structure and function. Among these works, a large portion of these methods are designed for ventricle segmentation, especially in MR and ultrasound domains. The objective of ventricle segmentation is to delineate the endocardium and epicardium of the LV and/or RV. These segmentation maps are important for deriving clinical indices, such as left ventricular end-diastolic volume (LVEDV), left ventricular end-systolic volume (LVESV), right ventricular end-diastolic volume (RVEDV), right ventricular end-systolic volume (RVESV), and EF. In addition, these segmentation maps are essential for 3D shape analysis (60, 86), 3D + time motion analysis (87), and survival prediction (88).

### 3.1. Cardiac MR Image Segmentation

Cardiac MRI is a non-invasive imaging technique that can visualize the structures within and around the heart. Compared to CT, it does not require ionizing radiation. Instead, it relies on the magnetic field in conjunction with radio-frequency waves to excite hydrogen nuclei in the heart, and then generates an image by measuring their response. By utilizing different imaging sequences, cardiac MRI allows accurate quantification of both cardiac anatomy and function (e.g., cine imaging) and pathological tissues, such as scars (late gadolinium enhancement (LGE) imaging). Accordingly, cardiac MRI is currently regarded as the gold standard for quantitative cardiac analysis (89).

A group of representative deep learning based cardiac MR segmentation methods are shown in Table 1. From the table, one can see that a majority of works have focused on segmenting cardiac chambers (e.g., LV, RV, LA). In contrast, there are relatively fewer works on segmenting abnormal cardiac tissue regions, such as myocardial scars and atrial fibrosis from contrast-enhanced images. This is likely due to the limited relevant public datasets as well as the difficulty of the task. In addition, to the best of our knowledge, there are very few works that apply deep learning techniques to atrial wall segmentation, as also suggested by a recent survey paper (161). In the following sections, we will describe and discuss these methods regarding different applications in detail.

**Table 1:** A summary of representative deep learning methods on cardiac MRI segmentation.

**Application**	**Selected works**	**Description**	**Type of images**	**Structure(s)**
	**FCN-based**			
	Tran (24)	2D FCN	SAX	Bi-ventricle
	Lieman-Sifry et al. (90)	A lightweight FCN (E-Net)	SAX	Bi-ventricle
	Isensee et al. (26)	2D U-net +3D U-net (ensemble)	SAX	Bi-ventricle
	Jang et al. (80)	2D M-Net with weighted cross entropy loss	SAX	Bi-ventricle
	Baumgartner et al. (25)	2D U-net with cross entropy	SAX	Bi-ventricle
	Bai et al. (31)	2D FCN trained and verified on a large dataset (~5000 subjects);	SAX, 2CH, 4CH	Four chambers
	Tao et al. (53)	2D U-net trained and verified on a multi-vendor, multi-scanner dataset	SAX	LV, Myo
	Khened et al. (29)	2D Dense U-net with inception module	SAX	Bi-ventricle
	Fahmy et al. (91)	2D FCN	SAX	LV, Myo
	**Introducing spatial or temporal context**			
	Poudel et al. (55)	2D FCN with RNN to model inter-slice coherency	SAX	Bi-ventricle
	Patravali et al. (92)	2D multi-channel FCN to aggregate inter-slice information	SAX	Bi-ventricle
Ventricle segmentation	Wolterink et al. (93)	Dilated U-net to segment ED and ES simultaneously	SAX	Bi-ventricle
	**Applying anatomical constraints**			
	Oktay et al. (59)	FCN trained with additional anatomical shape-based regularization	SAX; Ultrasound	LV, Myo
	**Multi-stage networks**			
	Tan et al. (94)	Semi-automated method; CNN (localization) followed by another CNN to derive contour parameters	SAX	LV, Myo
	Zheng et al. (27)	FCN (localization) + FCN (segmentation); Propagate labels from adjacent slices	SAX	Bi-ventricle
	Vigneault et al. (95)	U-net (initial segmentation) + CNN (localization and transformation) + Cascaded U-net (segmentation)	SAX, 2CH, 4CH	Four chambers
	**Hybrid segmentation methods**			
	Avendi et al. (47, 96)	CNN (localization) + AE (shape initialization) + Deformable model	SAX	LV, Myo/RV
	Yang et al. (97)	CNN combined with Multi-atlas	SAX	LV, Myo
	Ngo et al. (98)	Level-set based segmentation with deep belief networks	SAX	LV, Myo
	Mortazi et al. (99)	Multi-view CNN with adaptive fusion strategy	3D scans	LA
Atrial segmentation	Xiong et al. (100)	Patch-based dual-stream 2D FCN	LGE MRI	LA
	Xia et al. (54)	Two-stage pipeline; 3D U-net (localization) + 3D U-net (segmentation)	LGE MRI	LA
Scar segmentation	Yang et al. (101)	Fully automated; Multi-atlas method for LA segmentation followed by an AE to find the atrial scars	LGE MRI	LA; atrial scars
	Chen et al. (102)	Fully automated; Multi-view two-task recursive attention model	LGE MRI	LA; atrial scars
	Zabihollahy et al. (103)	Semi-automated; 2D CNN for scar tissue classification	LGE MRI	Myocardial scars
	Moccia et al. (104)	Semi-automated; 2D FCN for scar segmentation	LGE MRI	Myocardial scars
	Xu et al. (105)	Fully automated; RNN for joint motion feature learning and scar segmentaion	cine MRI	Myocardial scars
Aorta segmentation	Bai et al. (32)	RNN to learn temporal coherence; Propagate labels from labeled frames to unlabeled adjacent frames for semi-supervised learning;	cine MRI	Aorta
Whole heart segmentation	Yu et al. (30)	3D U-net with deep supervision	3D scans	Blood pool + Myocardium of the heart
	Li et al. (106)	3D FCN with deep supervision	3D scans	Blood pool + Myocardium of the heart
	Wolterink et al. (107)	dilated CNN with deep supervision	3D scans	Blood pool + Myocardium of the heart

*By default, LV/RV and LA/RA segmentation refer to the left/right ventricle cavity segmentation and left/right atrium cavity segmentation, respectively. The same applies to Tables 2**–**5. SAX, short-axis view; 2CH, 2-chamber view; 4CH, 4-chamber view; ED, end-diastolic; ES, end-systolic; Myo, Left ventricular myocardium*.

#### 3.1.1. Ventricle Segmentation

##### 3.1.1.1. Vanilla FCN-based segmentation

Tran (24) was among the first ones to apply a FCN (50) to segment the left ventricle, myocardium and right ventricle directly on short-axis cardiac magnetic resonance (MR) images. Their end-to-end approach based on FCN achieved competitive segmentation performance, significantly outperforming traditional methods in terms of both speed and accuracy. In the following years, a number of works based on FCNs have been proposed, aiming at achieving further improvements in segmentation performance. In this regard, one stream of work focuses on optimizing the network structure to enhance the feature learning capacity for segmentation (29, 80, 91, 162–165). For example, Khened et al. (29) developed a dense U-net with inception modules to combine multi-scale features for robust segmentation across images with large anatomical variability. Jang et al. (80), Yang et al. (81), Sander et al. (166), and Chen et al. (167) investigated different loss functions, such as weighted cross-entropy, weighted Dice loss, deep supervision loss and focal loss to improve the segmentation performance. Among these FCN-based methods, the majority of approaches use 2D networks rather than 3D networks for segmentation. This is mainly due to the typical low through-plane resolution and motion artifacts of most cardiac MR scans, which limits the applicability of 3D networks (25).

##### 3.1.1.2. Introducing spatial or temporal context

One drawback of using 2D networks for cardiac segmentation is that these networks work slice by slice, and thus they do not leverage any inter-slice dependencies. As a result, 2D networks can fail to locate and segment the heart on challenging slices, such as apical and basal slices where the contours of the ventricles are not well-defined. To address this problem, a number of works have attempted to introduce additional contextual information to guide 2D FCN. This contextual information can include shape priors learned from labels or multi-view images (109, 110, 168). Others extract spatial information from adjacent slices to assist the segmentation, using recurrent units (RNNs) or multi-slice networks (2.5D networks) (27, 55, 92, 169). These networks can also be applied to leveraging information across different temporal frames in the cardiac cycle to improve spatial and temporal consistency of segmentation results (28, 93, 169–171).

##### 3.1.1.3. Applying anatomical constraints

Another problem that may limit the segmentation performance of both 2D and 3D FCNs is that they are typically trained with pixel-wise loss functions only (e.g., cross-entropy or soft-Dice losses). These pixel-wise loss functions may not be sufficient to learn features that represent the underlying anatomical structures. Several approaches therefore focus on designing and applying anatomical constraints to train the network to improve its prediction accuracy and robustness. These constraints are represented as regularization terms which take into account the topology (172), contour and region information (173), or shape information (59, 63), encouraging the network to generate more anatomically plausible segmentations. In addition to regularizing networks at training time (61), proposed a variational AE to correct inaccurate segmentations, at the post-processing stage.

##### 3.1.1.4. Multi-task learning

Multi-task learning has also been explored to regularize FCN-based cardiac ventricle segmentation during training by performing auxiliary tasks that are relevant to the main segmentation task, such as motion estimation (174), estimation of cardiac function (175), ventricle size classification (176), and image reconstruction (177–179). Training a network for multiple tasks simultaneously encourages the network to extract features which are useful across these tasks, resulting in improved learning efficiency and prediction accuracy.

##### 3.1.1.5. Multi-stage networks

Recently, there is a growing interest in applying neural networks in a multi-stage pipeline which breaks down the segmentation problem into subtasks (27, 94, 95, 108, 180). For example, Zheng et al. (27) and Li et al. (108) proposed a region-of-interest (ROI) localization network followed by a segmentation network. Likewise, Vigneault et al. (95) proposed a network called Omega-Net which consists of a U-net for cardiac chamber localization, a learnable transformation module to normalize image orientation and a series of U-nets for fine-grained segmentation. By explicitly localizing the ROI and by rotating the input image into a canonical orientation, the proposed method better generalizes to images with varying sizes and orientations.

##### 3.1.1.6. Hybrid segmentation methods

Another stream of work aims at combining neural networks with classical segmentation approaches, e.g., level-sets (98, 181), deformable models (47, 96, 182), atlas-based methods (97, 111), and graph-cut based methods (183). Here, neural networks are applied in the feature extraction and model initialization stages, reducing the dependency on manual interactions and improving the segmentation accuracy of the conventional segmentation methods deployed afterwards. For example, Avendi et al. (47) proposed one of the first DL-based methods for LV segmentation in cardiac short-axis MR images. The authors first applied a CNN to automatically detect the LV and then used an AE to estimate the shape of the LV. The estimated shape was then used to initialize follow-up deformable models for shape refinement. As a result, the proposed integrated deformable model converges faster than conventional deformable models and the segmentation achieves higher accuracy. In their later work, the authors extended this approach to segment RV (96). While these hybrid methods demonstrated better segmentation accuracy than previous non-deep learning methods, most of them still require an iterative optimization for shape refinement. Furthermore, these methods are often designed for one particular anatomical structure. As noted in the recent benchmark study (17), most state-of-the-art segmentation algorithms for bi-ventricle segmentation are based on end-to-end FCNs, which allows the simultaneous segmentation of the LV and RV.

To better illustrate these developments for cardiac ventricle segmentation from cardiac MR images, we collate a list of bi-ventricle segmentation methods that have been trained and tested on the Automated Cardiac Diagnosis Challenge (ACDC) dataset, reported in Table 2. For ease of comparison, we only consider those methods which have been evaluated on the same online test set (50 subjects). As the ACDC challenge organizers keep the online evaluation platform open to the public, our comparison not only includes the methods from the original challenge participants [summarized in the benchmark study paper from Bernard et al. (17)] but also three segmentation algorithms that have been proposed after the challenge [i.e., (61, 108, 109)]. From this comparison, one can see that top algorithms are the ensemble method proposed by Isensee et al. (26) and the two-stage method proposed by Li et al. (108), both of which are based on FCNs. In particular, compared to the traditional level-set method (112), both methods achieved considerably higher accuracy even for the more challenging segmentation of the left ventricular myocardium (Myo), indicating the power of deep learning based approaches.

**Table 2:** Segmentation accuracy of state-of-the-art segmentation methods verified on the cardiac bi-ventricular segmentation challenge (ACDC) dataset (17).

**Methods**	**Description**	**LV**	**Myo**	**RV**
Isensee et al. (26)	2D U-net + 3D U-net (ensemble)	**0.950**	**0.911**	0.923
Li et al. (108)	Two 2D FCNs for ROI detection and segmentation, respectively;	0.944	**0.911**	**0.926**
Zotti et al. (109)	2D GridNet-MD with registered shape prior	0.938	0.894	0.910
Khened et al. (29)	2D Dense U-net with inception module	0.941	0.894	0.907
Baumgartner et al. (25)	2D U-net with cross entropy loss	0.937	0.897	0.908
Zotti et al. (110)	2D GridNet with registered shape prior	0.931	0.890	0.912
Jang et al. (80)	2D M-Net with weighted cross entropy loss	0.940	0.885	0.907
Painchaud et al. (61)	FCN followed by an AE for shape correction	0.936	0.889	0.909
Wolterink et al. (93)	Multi-input 2D dilated FCN, segmenting paired ED and ES frames simultaneously	0.940	0.885	0.900
Patravali et al. (92)	2D U-net with a Dice loss	0.920	0.890	0.865
Rohé et al. (111)	Multi-atlas based method combined with 3D CNN for registration	0.929	0.868	0.881
Tziritas and Grinias (112)	Level-set + markov random field (MRF); *Non-deep learning method*	0.907	0.798	0.803
Yang et al. (81)	3D FCN with deep supervision	0.820	N/A	0.780

*All the methods were evaluated on the same test set (50 subjects). Bold numbers are the highest overall Dice values for the corresponding structure. LV, left ventricle cavity; RV, right ventricle cavity; Myo, left ventricular myocardium; ED, end-diastolic; ES, end-systolic. Last update: 2019.8.1*.

*Note that for simplicity, we report the average Dice scores for each structure over ED and ES phases. More detailed comparison for different phases can be found on the public leaderboard in the post-testing part (https://acdc.creatis.insa-lyon.fr) as well as corresponding published works in this table*.

#### 3.1.2. Atrial Segmentation

Atrial fibrillation (AF) is one of the most common cardiac electrical disorders, affecting around 1 million people in the UK^5^. Accordingly, atrial segmentation is of prime importance in the clinic, improving the assessment of the atrial anatomy in both pre-operative AF ablation planning and post-operative follow-up evaluations. In addition, the segmentation of atrium can be used as a basis for scar segmentation and atrial fibrosis quantification from LGE images. Traditional methods, such as region growing (184) and methods that employ strong priors [i.e., atlas-based label fusion (185) and non-rigid registration (186)] have been applied in the past for automated left atrium segmentation. However, the accuracy of these methods highly relies on good initialization and *ad-hoc* pre-processing methods, which limits the widespread adoption in the clinic.

Recently, Vigneault et al. (95) and Bai et al. (31) applied 2D FCNs to directly segment the LA and RA from standard 2D long-axis images, i.e., 2-chamber (2CH), 4-chamber (4CH) views. Notably, their networks can also be trained to segment ventricles from 2D short-axis stacks without any modifications to the network architecture. Likewise, Xiong et al. (100), Preetha et al. (187), Bian et al. (188), and Chen et al. (34) applied 2D FCNs to segment the atrium from 3D LGE images in a slice-by-slice fashion, where they optimized the network structure for enhanced feature learning. 3D networks (54, 189–192) and multi-view FCN (99, 193) have also been explored to capture 3D global information from 3D LGE images for accurate atrium segmentation.

In particular, Xia et al. (54) proposed a fully automatic two-stage segmentation framework which contains a first 3D U-net to roughly locate the atrial center from down-sampled images followed by a second 3D U-net to accurately segment the atrium in the cropped portions of the original images at full resolution. Their multi-stage approach is both memory-efficient and accurate, ranking first in the left atrium segmentation challenge 2018 (LASC'18) with a mean Dice score of 0.93 evaluated on a test set of 54 cases.

#### 3.1.3. Scar Segmentation

Scar characterization is usually performed using LGE MR imaging, a contrast-enhanced MR imaging technique. LGE MR imaging enables the identification of myocardial scars and atrial fibrosis, allowing improved management of myocardial infarction and atrial fibrillation (194). Prior to the advent of deep learning, scar segmentation was often performed using intensity thresholding-based or clustering methods which are sensitive to the local intensity changes (103). The main limitation of these methods is that they usually require the manual segmentation of the region of interest to reduce the search space and the computational costs (195). As a result, these semi-automated methods are not suitable for large-scale studies or clinical deployment.

Deep learning approaches have been combined with traditional segmentation methods for the purpose of scar segmentation: Yang et al. (101, 196) applied an atlas-based method to identify the left atrium and then applied deep neural networks to detect fibrotic tissue in that region. Relatively to end-to-end approaches, Chen et al. (102) applied deep neural networks to segment both the left atrium and the atrial scars. In particular, the authors employed a multi-view CNN with a recursive attention module to fuse features from complementary views for better segmentation accuracy. Their approach achieved a mean Dice score of 0.90 for the LA region and a mean Dice score of 0.78 for atrial scars.

In the work of Fahmy et al. (197), the authors applied a U-net based network to segment the myocardium and the scars at the same time from LGE images acquired from patients with hypertrophic cardiomyopathy (HCM), achieving a fast segmentation speed. However, the reported segmentation accuracy for the scar regions was relatively low (mean Dice: 0.58). Zabihollahy et al. (103) and Moccia et al. (104) instead adopted a semi-automated method which requires a manual segmentation of the myocardium followed by the application of a 2D network to differentiate scars from normal myocardium. They reported higher segmentation accuracy on their test sets (mean Dice >0.68). At the moment, fully-automated scar segmentation is still a challenging task since the infarcted regions in patients can lead to kinematic variabilities and abnormalities in those contrast-enhanced images. Interestingly, Xu et al. (105) developed an RNN which leverages motion patterns to automatically delineate myocardial infarction area from cine MR image sequences without contrast agents. Their method achieved a high overall Dice score of 0.90 when compared to the manual annotations on LGE MR images, providing a novel approach for infarction assessment.

#### 3.1.4. Aorta Segmentation

The segmentation of the aortic lumen from cine MR images is essential for accurate mechanical and hemodynamic characterization of the aorta. One common challenge for this task is the typical sparsity of the annotations in aortic cine image sequences, where only a few frames have been annotated. To address the problem, Bai et al. (32) applied a non-rigid image registration method (198) to propagate the labels from the annotated frames to the unlabeled neighboring ones in the cardiac cycle, effectively generating pseudo annotated frames that could be utilized for further training. This semi-supervised method achieved an average Dice metric of 0.96 for the ascending aorta and 0.95 for the descending aorta over a test set of 100 subjects. In addition, compared to a previous approach based on deformable models (199), their approach based on FCN and RNN can directly perform the segmentation task on a whole image sequence without requiring the explicit estimation of the ROI.

#### 3.1.5. Whole Heart Segmentation

Apart from the above mentioned segmentation applications which target one particular structure, deep learning can also be applied to segmenting the main substructures of the heart in 3D MR images (30, 106, 107, 200). An early work from Yu et al. (30) adopted a 3D dense FCN to segment the myocardium and blood pool in the heart from 3D MR scans. Recently, more and more methods began to apply deep learning pipelines to segment more specific substructures [including four chambers, aorta, pulmonary vein (PV)] in both 3D CT and MR images. This has been facilitated by the availability of a public dataset for whole heart segmentation [Multi-Modality Whole Heart Segmentation (MM-WHS)] which consists of both CT and MRI images. We will discuss these segmentation methods in the next CT section in further detail (see section 3.2.1).

### 3.2. Cardiac CT Image Segmentation

CT is a non-invasive imaging technique that is performed routinely for disease diagnosis and treatment planning. In particular, cardiac CT scans are used for the assessment of cardiac anatomy and specifically the coronary arteries. There are two main imaging modalities: non-contrast CT imaging and contrast-enhanced coronary CT angiography (CTA). Typically, non-contrast CT imaging exploits density of tissues to generate an image, such that different densities using various attenuation values, such as soft tissues, calcium, fat, and air can be easily distinguished, and thus allows to estimate the amount of calcium present in the coronary arteries (201). In comparison, contrast-enhanced coronary CTA, which is acquired after the injection of a contrast agent, can provide excellent visualization of cardiac chambers, vessels and coronaries, and has been shown to be effective in detecting non-calcified coronary plaques. In the following sections, we will review some of the most commonly used deep learning-based cardiac CT segmentation methods. A summary of these approaches is presented in Table 3.

**Table 3:** A summary of selected deep learning methods on cardiac CT segmentation.

**Application**	**Selected works**	**Description**	**Imaging modality**	**Structure(s)**
	**Two-step segmentation**			
	Zreik et al. (113)	Patch-based CNN	CTA	Myo
	Payer et al. (114)	A pipeline of two FCNs	MR/CT	WHS
	Tong et al. (115)	Deeply supervised 3D U-net	MR/CT	WHS
	Wang et al. (116)	Two-stage 3D U-net with dynamic ROI extraction	MR/CT	WHS
	Xu et al. (37)	Faster RCNN and U-net	CT	WHS
Cardiac substructure segmentation	**Multi-view CNNs**			
	Wang and Smedby (117)	Orthogonal 2D U-nets with shape context	MR/CT	WHS
	Mortazi et al. (118)	Multi-planar FCNs with an adaptive fusion strategy	MR/CT	WHS
	**Hybrid loss**			
	Yang et al. (36)	3D U-net with deep supervision	MR/CT	WHS
	Ye et al. (119)	3D deeply-supervised U-net with multi-depth fusion	CT	WHS
	**Others**			
	Zreik et al. (120)	Multi-scale FCN	CTA	Myo
	Joyce et al. (121)	Unsupervised segmentation with GANs	MR/CT	LV, Myo, RV
	**End-to-end CNNs**			
	Moeskops et al. (122)	Multi-task CNN	CTA	Vessel
	Merkow et al. (38)	3D U-net with deep multi-scale supervision	CTA	Vessel
	Lee et al. (123)	Template transformer network	CTA	Vessel
	**CNN as pre-/post-processing**			
Coronary artery segmentation	Gülsün et al. (124)	CNN as path pruning	CTA	coronary artery centerline
	Guo et al. (125)	Multi-task FCN with a minimal patch extractor	CTA	Coronary artery centerline
	Shen et al. (126)	3D FCN with level set	CTA	Vessel
	**Others**			
	Wolterink et al. (127)	CNN to estimate direction classification and radius regression	CTA	Coronary artery centerline
	Wolterink et al. (128)	Graph convolutional network	CTA	Vessel
	**Two-step segmentation**			
	Wolterink et al. (129)	CNN pairs	CTA	CAC
	Lessmann et al. (130)	Multi-view CNNs	CT	CAC
	Lessmann et al. (131)	Two consecutive CNNs	CT	CAC
	Liu et al. (132)	3D vessel-focused ConvNets	CTA	CAC, NCP, MCP
Coronary artery calcium and plaque segmentation	**Direct segmentation**			
	Santini et al. (133)	Patch-based CNN	CT	CAC
	Shadmi et al. (134)	U-net and FC DenseNet	CT	CAC
	Zhang et al. (135)	U-DenseNet	CT	CAC
	Ma and Zhang (136)	DenseRAU-net	CT	CAC

#### 3.2.1. Cardiac Substructure Segmentation

Accurate delineation of cardiac substructures plays a crucial role in cardiac function analysis, providing important clinical variables, such as EF, myocardial mass, wall thickness etc. Typically, the cardiac substructures that are segmented include the LV, RV, LA, RA, Myo, aorta (AO), and pulmonary artery (PA).

##### 3.2.1.1. Two-step segmentation

One group of deep learning methods relies on a two-step segmentation procedure, where a ROI is first extracted and then fed into a CNN for subsequent classification (113, 202). For instance, Zreik et al. (113) proposed a two-step LV segmentation process where a bounding box for the LV is first detected using the method described in de Vos et al. (203), followed by a voxel classification within the defined bounding box using a patch-based CNN. More recently, FCN, especially U-net (49), has become the method of choice for cardiac CT segmentation. Zhuang et al. (19) provides a comparison of a group of methods (36, 114, 115, 117, 118, 137) for whole heart segmentation (WHS) that have been evaluated on the MM-WHS challenge. Several of these methods (37, 114–116) combine a localization network, which produces a coarse detection of the heart, with 3D FCNs applied to the detected ROI for segmentation. This allows the segmentation network to focus on the anatomically relevant regions, and has shown to be effective for whole heart segmentation. A summary of the comparison between the segmentation accuracy of the methods evaluated on MM-WHS dataset is presented in Table 4. These methods generally achieve better segmentation accuracy on CT images compared to that of MR images, mainly because of the smaller variations in image intensity distribution across different CT scanners and better image quality (19). For a detailed discussion on these listed methods, please refer to Zhuang et al. (19).

**Table 4:** Segmentation accuracy of methods validated on MM-WHS dataset.

**Methods**	**LV**	**RV**	**LA**	**RA**	**Myo**	**AO**	**PA**	**WHS**
Payer et al. (114)	91.8/91.6	**90.9**/86.8	92.9/85.5	**88.8**/88.1	88.1/77.8	93.3/88.8	84.0/73.1	**90.8**/86.3
Yang et al. (137)	92.3/75.0	85.7/75.0	**93.0**/82.6	87.1/85.9	85.6/65.8	89.4/80.9	83.5/72.6	89.0/78.3
Mortazi et al. (118)	90.4/87.1	88.3/83.0	91.6/81.1	83.6/75.9	85.1/74.7	90.7/83.9	78.4/71.5	87.9/81.8
Tong et al. (115)	89.3/70.2	81.0/68.0	88.9/67.6	81.2/65.4	83.7/62.3	86.8/59.9	69.8/47.0	84.9/67.4
Wang et al. (116)	80.0/86.3	78.6/84.9	90.4/85.2	79.4/84.0	72.9/74.4	87.4/82.4	64.8/78.8	80.6/83.2
Ye et al. (119)	**94.4**/–	89.5/–	91.6/–	87.8/–	**88.9**/–	**96.7**/–	**86.2**/–	90.7/–
Xu et al. (37)	87.9/–	90.2/–	83.2/–	84.4/–	82.2/–	91.3/–	82.1/–	85.9/–

*The training set contains 20 CT and 20 MRI whereas the test set contains 40 CT and 40 MRI. Reported numbers are Dice scores (CT/MRI) for different substructures on both CT and MRI scans. For more detailed comparisons, please refer to Zhuang et al. (19). The bold number in each column represents the highest score for the corresponding structure on CT images*.

##### 3.2.1.2. Multi-view CNNs

Another line of research utilizes the volumetric information of the heart by training multi-planar CNNs (axial, sagittal, and coronal views) in a 2D fashion. Examples include Wang et al. (117) and Mortazi et al. (118) where three independent orthogonal CNNs were trained to segment different views. Specifically, Wang et al. (117) additionally incorporated shape context in the framework for the segmentation refinement, while Mortazi et al. (118) adopted an adaptive fusion strategy to combine multiple outputs utilizing complementary information from different planes.

##### 3.2.1.3. Hybrid loss

Several methods employ a hybrid loss, where different loss functions (such as focal loss, Dice loss, and weighted categorical cross-entropy) are combined to address the class imbalance issue, e.g., the volume size imbalance among different ventricular structures, and to improve the segmentation performance (36, 119).

In addition, the work of Zreik et al. (120) has proposed a method for the automatic identification of patients with significant coronary artery stenoses through the segmentation and analysis of the LV myocardium. In this work, a multi-scale FCN is first employed for myocardium segmentation, and then a convolutional autoencoder is used to characterize the LV myocardium, followed by a support vector machine (SVM) to classify patients based on the extracted features.

#### 3.2.2. Coronary Artery Segmentation

Quantitative analysis of coronary arteries is an important step for the diagnosis of cardiovascular diseases, stenosis grading, blood flow simulation and surgical planning (204). Though this topic has been studied for years (4), only a small number of works investigate the use of deep learning in this context. Methods relating to coronary artery segmentation can be mainly divided into two categories: centerline extraction and lumen (i.e., vessel wall) segmentation.

##### 3.2.2.1. CNNs as a post-/pre-processing step

Coronary centerline extraction is a challenging task due to the presence of nearby cardiac structures and coronary veins as well as motion artifacts in cardiac CT. Several deep learning approaches employ CNNs as either a post-processing or pre-processing step for traditional methods. For instance, Gülsün et al. (124) formulated centerline extraction as finding the maximum flow paths in a steady state porous media flow, with a learning-based classifier estimating anisotropic vessel orientation tensors for flow computation. A CNN classifier was then employed to distinguish true coronary centerlines from leaks into non-coronary structures. Guo et al. (125) proposed a multi-task FCN centerline extraction method that can generate a single-pixel-wide centerline, where the FCN simultaneously predicted centerline distance maps and endpoint confidence maps from coronary arteries and ascending aorta segmentation masks, which were then used as input to the subsequent minimal path extractor to obtain the final centerline extraction results. In contrast, unlike the aforementioned methods that used CNNs either as a pre-processing or post-processing step, Wolterink et al. (127) proposed to address centerline extraction via a 3D dilated CNN, where the CNN was trained on patches to directly determine a posterior probability distribution over a discrete set of possible directions as well as to estimate the radius of an artery at the given point.

##### 3.2.2.2. End-to-end CNNs

With respect to the lumen or vessel wall segmentation, most deep learning based approaches use an end-to-end CNN segmentation scheme to predict dense segmentation probability maps (38, 122, 126, 205). In particular, Moeskops et al. (122) proposed a multi-task segmentation framework where a single CNN can be trained to perform three different tasks including coronary artery segmentation in cardiac CTA and tissue segmentation in brain MR images. They showed that such a multi-task segmentation network in multiple modalities can achieve equivalent performance as a single task network. Merkow et al. (38) introduced deep multi-scale supervision into a 3D U-net architecture, enabling efficient multi-scale feature learning and precise voxel-level predictions. Besides, shape priors can also be incorporated into the network (123, 206, 207). For instance, Lee et al. (123) explicitly enforced a roughly tubular shape prior for the vessel segments by introducing a template transformer network, through which a shape template can be deformed via network-based registration to produce an accurate segmentation of the input image, as well as to guarantee topological constraints. More recently, graph convolutional networks have also been investigated by Wolterink et al. (128) for coronary artery segmentation in CTA, where vertices on the coronary lumen surface mesh were considered as graph nodes and the locations of these tubular surface mesh vertices were directly optimized. They showed that such method significantly outperformed a baseline network that used only fully-connected layers on healthy subjects (mean Dice score: 0.75 vs. 0.67). Besides, the graph convolutional network used in their work is able to directly generate smooth surface meshes without post-processing steps.

#### 3.2.3. Coronary Artery Calcium and Plaque Segmentation

Coronary artery calcium (CAC) is a direct risk factor for cardiovascular disease. Clinically, CAC is quantified using the Agatston score (208) which considers the lesion area and the weighted maximum density of the lesion (209). Precise detection and segmentation of CAC are thus important for the accurate prediction of the Agatston score and disease diagnosis.

##### 3.2.3.1. Two-step segmentation

One group of deep learning approaches to segmentation and automatic calcium scoring proposed to use a two-step segmentation scheme. For example, Wolterink et al. (129) attempted to classify CAC in cardiac CTA using a pair of CNNs, where the first CNN coarsely identified voxels likely to be CAC within a ROI detected using De et al. (203) and then the second CNN further distinguished between CAC and CAC-like negatives more accurately. Similar to such a two-stage scheme, Lessmann et al. (130, 131) proposed to identify CAC in low-dose chest CT, in which a ROI of the heart or potential calcifications were first localized followed by a CAC classification process.

##### 3.2.3.2. Direct segmentation

More recently, several approaches (133–136) have been proposed for the direct segmentation of CAC from non-contrast cardiac CT or chest CT: the majority of them employed combinations of U-net (49) and DenseNet (78) for precise quantification of CAC which showed that a sensitivity over 90% can be achieved (133). These aforementioned approaches all follow the same workflow where the CAC is first identified and then quantified. An alternative approach is to circumvent the intermediate segmentation and to perform direct quantification, such as in de Vos et al. (209) and Cano-Espinosa et al. (210), which have proven that this approach is effective and promising.

Finally, for non-calcified plaque (NCP) and mixed-calcified plaque (MCP) in coronary arteries, only a limited number of works have been reported that investigate deep learning methods for segmentation and quantification (132, 211). Yet, this is a very important task from a clinical point of view, since these plaques can potentially rupture and obstruct an artery, causing ischemic events and severe cardiac damage. In contrast to CAC segmentation, NCP and MCP segmentation are more challenging due to their similar appearances and intensities as adjacent tissues. Therefore, robust and accurate analysis often requires the generation of multi-planar reformatted (MPR) images that have been straightened along the centerline of the vessel. Recently, Liu et al. (132) proposed a vessel-focused 3D convolutional network with attention layers to segment three types of plaques on the extracted and reformatted coronary MPR volumes. Zreik et al. (211) presented an automatic method for detection and characterization of coronary artery plaques as well as determination of coronary artery stenosis significance, in which a multi-task convolutional RNN was used to perform both plaque and stenosis classification by analyzing the features extracted along the coronary artery in an MPR image.

### 3.3. Cardiac Ultrasound Image Segmentation

Cardiac ultrasound imaging, also known as echocardiography, is an indispensable clinical tool for the assessment of cardiovascular function. It is often used clinically as the first imaging examination owing to its portability, low cost and real-time capability. While a number of traditional methods, such as active contours, level-sets and active shape models have been employed to automate the segmentation of anatomical structures in ultrasound images (212), the achieved accuracy is limited by various problems of ultrasound imaging, such as low signal-to-noise ratio, varying speckle noise, low image contrast (especially between the myocardium and the blood pool), edge dropout and shadows cast by structures, such as dense muscle and ribs.

As in cardiac MR and CT, several DL-based methods have been recently proposed to improve the performance of cardiac ultrasound image segmentation in terms of both accuracy and speed. The majority of these DL-based approaches focus on LV segmentation, with only few addressing the problem of aortic valve and LA segmentation. A summary of the reviewed works can be found in Table 5.

**Table 5:** A summary of reviewed deep learning methods for ultrasound image segmentation.

**Application**	**Selected works**	**Method**	**Structure**	**Imaging modality**
	**Combined with deformable models**			
	Carneiro et al. (138, 139)	DBN with two-step approach: localization and fine segmentation	LV	2D A2C, A4C
	Nascimento and Carneiro (140)	deep belief networks (DBN) and sparse manifold learning for the localization step	LV	2D A2C, A4C
	Nascimento and Carneiro (141, 142)	DBN and sparse manifold learning for one-step segmentation	LV	2D A2C, A4C
	Veni et al. (143)	FCN (U-net) followed by level-set based deformable model	LV	2D A4C
	**Utilizing temporal coherence**			
2D LV	Carneiro and Nascimento (144, 145)	DBN and particle filtering for dynamic modeling	LV	2D A2C, A4C
	Jafari et al. (146)	U-net and LSTM with additional optical flow input	LV	2D A4C
	**Utilizing unlabeled data**			
	Carneiro and Nascimento (147, 148)	DBN on-line retrain using external classifier as additional supervision	LV	2D A2C, A4C
	Smistad et al. (149)	U-Net trained using labels generated by a Kalman filter based method	LV and LA	2D A2C, A4C
	Yu et al. (150)	Dynamic CNN fine-tuning with mitral valve tracking to separate LV from LA	Fetal LV	2D
	Jafari et al. (151)	U-net with TL-net (152) based shape constraint on unannotated frames	LV	2D A4C
	**Utilizing data from multiple domains**			
	Chen et al. (153)	FCN trained using annotated data of multiple anatomical structures	Fetal head and LV	2D head, A2-5C
	**Others**			
	Smistad et al. (154)	Real time CNN view-classification and segmentation	LV	2D A2C, A4C
	Leclerc et al. (155)	U-net trained on a large heterogeneous dataset	LV, Myo	2D A4C
	Jafari et al. (156)	Real-time mobile software, lightweight U-Net, multitask and adversarial training	LV	2D A2C, A4C
	Dong et al. (157)	CNN for 2D coarse segmentation refined by 3D snake model	LV	3D (CETUS)
3D LV	Oktay et al. (59)	U-net with TL-net based shape constraint	LV	3D (CETUS)
	Dong et al. (158)	Atlas-based segmentation using DL registration and adversarial training	LV	3D
	Ghesu et al. (159)	Marginal space learning and adaptive sparse neural network	Aortic valves	3D
Others	Degel et al. (160)	V-net with TL-net based shape constraint and GAN-based domain adaptation	LA	3D
	Zhang et al. (42)	CNN for view-classification, segmentation and disease detection	Multi-chamber	2D PLAX, PSAX, A2-4C

*A[X]C is short for Apical [X]-chamber view. PLAX/PSAX, parasternal long-axis/short-axis; CETUS, using the dataset from Challenge on Endocardial Three-dimensional Ultrasound Segmentation*.

#### 3.3.1. 2D LV Segmentation

##### 3.3.1.1. Deep learning combined with deformable models

The imaging quality of echocardiography makes voxel-wise tissue classification highly challenging. To address this challenge, deep learning has been combined with deformable model for LV segmentation in 2D images (138, 139, 141–145). Features extracted by trained deep neural networks were used instead of handcrafted features to improve accuracy and robustness.

Several works applied deep learning in a two-stage pipeline which first localizes the target ROI via rigid transformation of a bounding box, then segments the target structure within the ROI. This two-stage pipeline reduces the search region of the segmentation and increases robustness of the overall segmentation framework. Carneiro et al. (138, 139) first adopted this DL framework to segment the LV in apical long-axis echocardiograms. The method uses DBN (213) to predict the rigid transformation parameters for localization and the deformable model parameters for segmentation. The results demonstrated the robustness of DBN-based feature extraction to image appearance variations. Nascimento and Carneiro (140) further reduced the training and inference complexity of the DBN-based framework by using sparse manifold learning in the rigid detection step.

To further reduce the computational complexity, some works perform segmentation in one step without resorting to the two-stage approach. Nascimento and Carneiro (141, 142) applied sparse manifold learning in segmentation, showing a reduced training and search complexity compared to their previous version of the method, while maintaining the same level of segmentation accuracy. Veni et al. (143) applied a FCN to produce coarse segmentation masks, which is then further refined by a level-set based method.

##### 3.3.1.2. Utilizing temporal coherence

Cardiac ultrasound data is often recorded as a temporal sequence of images. Several approaches aim to leverage the coherence between temporally close frames to improve the accuracy and robustness of the LV segmentation. Carneiro and Nascimento (144, 145) proposed a dynamic modeling method based on a sequential monte carlo (SMC) (or particle filtering) framework with a transition model, in which the segmentation of the current cardiac phase depends on previous phases. The results show that this approach performs better than the previous method (138) which does not take temporal information into account. In a more recent work, Jafari et al. (146) combined U-net, long-short term memory (LSTM) and inter-frame optical flow to utilize multiple frames for segmenting one target frame, demonstrating improvement in overall segmentation accuracy. The method was also shown to be more robust to image quality variations in a sequence than single-frame U-net.

##### 3.3.1.3. Utilizing unlabeled data

Several works proposed to use non-DL based segmentation algorithms to help generating labels on unlabeled images, effectively increasing the amount of training data. To achieve this, Carneiro and Nascimento (147, 148) proposed on-line retraining strategies where segmentation network (DBN) is firstly initialized using a small set of labeled data and then applied to non-labeled data to propose annotations. The proposed annotations are then checked by external classifiers before being used to re-train the network. Smistad et al. (149) trained a U-net using images annotated by a Kalman filtering based method (214) and illustrated the potential of using this strategy for pre-training. Alternatively, some works proposed to exploit unlabeled data without using additional segmentation algorithm. Yu et al. (150) proposed to train a CNN on a partially labeled dataset of multiple sequences, then fine-tuned the network for each individual sequence using manual segmentation of the first frame as well as CNN-produced label of other frames. Jafari et al. (151) proposed a semi-supervised framework which enables training on both the labeled and unlabeled images. The framework uses an additional generative network, which is trained to generate ultrasound images from segmentation masks, as additional supervision for the unlabeled frames in the sequences. The generative network forces the segmentation network to predict segmentation that can be used to successfully generate the input ultrasound image.

##### 3.3.1.4. Utilizing data from multiple domains

Apart from exploiting unlabeled data in the same domain, leveraging manually annotated data from multiple domains (e.g., different 2D ultrasound views with various anatomical structures) can also help to improve the segmentation in one particular domain. Chen et al. (153) proposed a novel FCN-based network to utilize multi-domain data to learn generic feature representations. Combined with an iterative refinement scheme, the method has shown superior performance in detection and segmentation over traditional database-guided method (215), FCN trained on single-domain and other multi-domain training strategies.

##### 3.3.1.5. Others

The potential of CNN in segmentation has motivated the collection and labeling of large-scale datasets. Several methods have since shown that deep learning methods, most notably CNN-based methods, are capable of performing accurate segmentation directly without complex modeling and post-processing. Leclerc et al. (155) performed a study to investigate the effect of the size of annotated data for the segmentation of the LV in 2D ultrasound images using a simple U-net. The authors demonstrated that the U-net approach significantly benefits from larger amounts of training data. In addition to performance on accuracy, some work investigated the computational efficiency of DL-based methods. Smistad et al. (154) demonstrated the efficiency of CNN-based methods by successfully performing real-time view-classification and segmentation. Jafari et al. (156) developed a software pipeline capable of real-time automated LV segmentation, landmark detection and LV ejection fraction calculation on a mobile device taking input from point-of-care ultrasound (POCUS) devices. The software uses a lightweight U-net trained using multi-task learning and adversarial training, which achieves EF prediction error that is lower than inter- and intra- observer variability.

#### 3.3.2. 3D LV Segmentation

Segmenting cardiac structures in 3D ultrasound is even more challenging than 2D. While having the potential to derive more accurate volume-related clinical indices, 3D echocardiograms suffer from lower temporal resolution and lower image quality compared to 2D echocardiograms. Moreover, 3D images dramatically increase the dimension of parameter space of neural networks, which poses computational challenges for deep learning methods.

One way to reduce the computational cost is to avoid direct processing of 3D data in deep learning networks. Dong et al. (157) proposed a two-stage method by first applying a 2D CNN to produce coarse segmentation maps on 2D slices from a 3D volume. The coarse 2D segmentation maps are used to initialize a 3D shape model which is then refined by 3D deformable model method (216). In addition, the authors used transfer learning to side-step the limited training data problem by pre-training network on a large natural image segmentation dataset and then fine-tuning to the LV segmentation task.

Anatomical shape priors have been utilized to increase the robustness of deep learning-based segmentation methods to challenging 3D ultrasound images. Oktay et al. (59) proposed an anatomically constrained network where a shape constraint-based loss is introduced to train a 3D segmentation network. The shape constraint is based on the shape prior learned from segmentation maps using auto-encoders (152). Dong et al. (158) utilized shape prior more explicitly by combining a neural network with a conventional atlas-based segmentation framework. Adversarial training was also applied to encourage the method to produce more anatomically plausible segmentation maps, which contributes to its superior segmentation performance comparing to a standard voxel-wise classification 3D segmentation network (52).

#### 3.3.3. Left Atrium Segmentation

Degel et al. (160) adopted the aforementioned anatomical constraints in 3D LA segmentation to tackle the domain shift problem caused by variation of imaging device, protocol and patient condition. In addition to the anatomically constraining network, the authors applied an adversarial training scheme (217) to improve the generalizability of the model to unseen domain.

#### 3.3.4. Multi-Chamber Segmentation

Apart from LV segmentation, a few works (23, 42, 149) applied deep learning methods to perform multi-chamber (including LV and LA) segmentation. In particular, (42) demonstrated the applicability of CNNs on three tasks: view classification, multi-chamber segmentation and detection of cardiovascular diseases. Comprehensive validation on a large (non-public) clinical dataset showed that clinical metrics derived from automatic segmentation are comparable or superior than manual segmentation. To resemble real clinical situations and thus encourages the development and evaluation of robust and clinically effective segmentation methods, a large-scale dataset for 2D cardiac ultrasound has been recently made public (23). The dataset and evaluation platform were released following the preliminary data requirement investigation of deep learning methods (155). The dataset is composed of apical 4-chamber view images annotated for LV and LA segmentation, with uneven imaging quality from 500 patients with varying conditions. Notably, the initial benchmarking (23) on this dataset has shown that modern encoder-decoder CNNs resulted in lower error than inter-observer error between human cardiologists.

#### 3.3.5. Aortic Valve Segmentation

Ghesu et al. (159) proposed a framework based on marginal space learning (MSL), Deep neural networks (DNNs) and active shape model (ASM) to segment the aortic valve in 3D cardiac ultrasound volumes. An adaptive sparsely-connected neural network with reduced number of parameters is used to predict a bounding box to locate the target structure, where the learning of the bounding box parameters is marginalized into sub-spaces to reduce computational complexity. This framework showed significant improvement over the previous non-DL MSL (218) method while achieving competitive run-time.

### 3.4. Discussion

So far, we have presented and discussed recent progress of deep learning-based segmentation methods in the three modalities (i.e., MR, CT, ultrasound) that are commonly used in the assessment of cardiovascular disease. To summarize, current state-of-the-art segmentation methods are mainly based on CNNs that employ the FCN or U-net architecture. In addition, there are several commonalities in the FCN-based methods for cardiac segmentation which can be categorized into four groups: (1) enhancing network feature learning by employing advanced building blocks in networks (e.g., inception module, dilated convolutions), most of which have been mentioned earlier (section 2.1.6); (2) alleviating the problem of class imbalance with advanced loss functions (e.g., weighted loss functions); (3) improving the networks' generalization ability and robustness through a multi-stage pipeline, multi-task learning, or multi-view feature fusion; (4) forcing the network to generate more anatomically-plausible segmentation results by incorporating shape priors, applying adversarial loss or anatomical constraints to regularize the network during training. It is also worthwhile to highlight that for cardiac image sequence segmentation (e.g., cine MR images, 2D ultrasound sequences), leveraging spatial and temporal coherence from these sequences with advanced neural networks [e.g., RNN (32, 146), multi-slice FCN (27)] has been explored and shown to be beneficial for improving the segmentation accuracy and temporal consistency of the segmentation maps.

While the results reported in the literature show that neural networks have become more sophisticated and powerful, it is also clear that performance has improved with the increase of publicly available training subjects. A number of DL-based methods (especially in MRI) have been trained and tested on public challenge datasets, which not only provide large amounts of data to exploit the capabilities of deep learning in this domain, but also a platform for transparent evaluation and comparison. In addition, many of the participants in these challenges have shared their code with other researchers via open-source community websites (e.g., Github). Transparent and fair benchmarking and sharing of code are both essential for continued progress in this domain. We summarize the existing public datasets in Table 6 and public code repositories in Table 7 for reference.

**Table 6:** Summary of public datasets on cardiac segmentation for the three modalities.

**Dataset Name/ References**	**Year**	**Main modalities**	**# of subjects**	**Target(s)**	**Main pathology**
York (10)	2008	cine MRI	33	LV, Myo	Cardiomyopathy, aortic regurgitation, enlarged ventricles and ischemia
Sunnybrook (11)	2009	cine MRI	45	LV, Myo	Hypertrophy, heart failure w./w.o infarction
LVSC (12)	2011	cine MRI	200	LV, Myo	Coronary artery disease, myocardial infarction.
RVSC (1)	2012	cine MRI	48	RV	Myocarditis, ischemic cardiomyopathy, suspicion of arrhythmogenic, right ventricular dysplasia, dilated cardiomyopathy, hypertrophic cardiomyopathy, aortic stenosis
cDEMRIS (13)	2012	LGE MRI	60	LA fibrosis and scar	Atrial fibrillation
LVIC (14)	2012	LGE MRI	30	Myocardial scars	Ischemic cardiomyopathy
LASC'13 (15)	2013	3D MRI	30	LA	N/A
HVSMR (16)	2016	3D MRI	4	Blood pool, myocardium of the heart	Congenital heart defects
ACDC (17)	2017	cine MRI	150	LV, Myo; RV	Mycardial infarction, dilated/hypertrophic cardiomyopathy, abnormal RV
LASC'18 (18)	2018	LGE MRI	154	LA	Atrial fibrillation
MM-WHS (19)	2017	CT/MRI	60/60	WHS	Myocardium infarction, atrial fibrillation, tricuspid regurgitation, aortic valve stenosis, Alagille syndrome, Williams syndrome, dilated cardiomyopathy, aortic coarctation, tetralogy of Fallot
CAT08 (20)	2008	CTA	32	Coronary artery centerline	Patients with presence of calcium scored as absent, modest or severe.
CLS12 (21)	2012	CTA	48	Coronary lumen and stenosis	Patients with different levels of coronary artery stenoses.
CETUS (22)	2014	3D Ultrasound	45	LV	Myocardial infarction, dilated cardiomyopathy
CAMUS (23)	2019	2D Ultrasound	500	LV, LA	Patients with EF <45%

*Most of the datasets listed above are from the International Conference on Medical Image Computing and Computer-assisted Intervention (MICCAI) society*.

**Table 7:** Public code for DL-based cardiac image segmentation.

**Modality**	**Application(s)**	**References**	**Basic network**	**Code repo (If not specified, the repository is located under github.com)**
MR (SAX)	Bi-ventricular Segmentation	Tran (24)	2D FCN	vuptran/cardiac-segmentation
MR (SAX)	Bi-ventricular Segmentation	Baumgartner et al. (25)	2D/3D U-net	baumgach/acdc_segmenter
MR (SAX)	Bi-ventricular Segmentation; 1st rank in ACDC challenge	Isensee et al. (26)	2D + 3D U-net (ensemble)	MIC-DKFZ/ACDC2017
MR (SAX)	Bi-ventricular Segmentation	Zheng et al. (27)	Cascaded 2D U-net	julien-zheng/CardiacSegmentationPropagation
MR (SAX)	Bi-ventricular segmentation and Motion Estimation	Qin et al. (28)	2D FCN, RNN	cq615
MR (SAX)	Biventricular Segmentation	Khened et al. (29)	2D U-net	mahendrakhened
MR (3D scans)	WHS	Yu et al. (30)	3D CNN	yulequan/HeartSeg
MR (Multi-view)	Four-chamber Segmentation and Aorta Segmentation	Bai et al. (31, 32)	2D FCN, RNN	baiwenjia/ukbb_cardiac
MR	Cardiac segmentation and motion tracking	Duan et al. (33)	2.5D FCN + Atlas-based	j-duan/4Dsegment
LGE MRI	Left Atrial Segmentation	Chen et al. (34)	2D U-net	cherise215/atria_segmentation_2018
LGE MRI	Left Atrial Segmentation	Yu et al. (35)	3D V-net	yulequan/UA-MT
CT	WHS	Yang et al. (36)	3D U-net	xy0806/miccai17-mmwhs-hybrid
CT	WHS	Xu et al. (37)	Faster RCNN, 3D U-net	Wuziyi616/CFUN
CT, MRI	Coronary arteries	Merkow et al. (38)	3D U-net	jmerkow/I2I
CT, MRI	WHS	Dou et al. (39, 40)	2D CNN	carrenD/Medical-Cross-Modality-Domain-Adaptation
CT, MRI	WHS	Chen et al. (41)	2D CNN	cchen-cc/SIFA
Ultrasound	View classification and four-chamber segmentation	Zhang et al. (42)	2D U-net	bitbucket.org/rahuldeo/echocv

*SAX, short-axis view; WHS, whole heart segmentation*.

An interesting conclusion supported by Table 7 is that the target image type can affect the choice of network structures (i.e., 2D networks, 3D networks). For 3D imaging acquisitions, such as LGE-MRI and CT images, 3D networks are preferred whereas 2D networks are more popular approaches for segmenting cardiac cine short-axis or long-axis image stacks. One reason for using 2D networks for the segmentation of short-axis or long-axis images is their typically large slice thickness (usually around 7–8 mm) which can further exacerbated by inter-slice gaps. In addition, breath-hold related motion artifacts between different slices may negatively affect 3D networks. A study conducted by Baumgartner et al. (25) has shown that a 3D U-net performs worse than a 2D U-net when evaluated on the ACDC challenge dataset. By contrast, in the LASC'18 challenge mentioned in Table 6, which uses high-resolution 3D images, most participants applied 3D networks and the best performance was achieved by a cascaded network based on the 3D U-net (54).

It is well-known that training 3D networks is more difficult than training 2D networks. In general, 3D networks have significantly more parameters than 2D networks. Therefore, 3D networks are more difficult and computationally expensive to optimize as well as prone to over-fitting, especially if the training data is limited. As a result, several researchers have tried to carefully design the structure of network to reduce the number of parameters for a particular application and have also applied advanced techniques (e.g., deep supervision) to alleviate the over-fitting problem (30, 54). For this reason, 2D-based networks (e.g., 2D U-net) are still the most popular segmentation approaches for all three modalities.

In addition to 2D and 3D networks, several authors have proposed “2D+” networks that have been shown to be effective in segmenting structures from cardiac volumetric data. These “2D+” networks are mainly based on 2D networks, but are adapted with increased capacity to utilize 3D context. These networks include multi-view networks which leverage multi-planar information (i.e., coronal, sagittal, axial views) (99, 117), multi-slice networks, and 2D FCNs combined with RNNs which incorporate context across multiple slices (33, 55, 92, 169). These “2D+” networks inherit the advantages of 2D networks while still being capable of leveraging through-plane spatial context for more robust segmentation with strong 3D consistency.

Finally, it is worth to note that there is no universally optimal segmentation method. Different applications have different complexities and different requirements, meaning that customized algorithms need to be optimized. For example, while anatomical shape constraints can be applied to cardiac anatomical structure segmentation (e.g., ventricle segmentation) to boost the segmentation performance, those constraints may not be suitable for the segmentation of pathologies or lesions (e.g., scar segmentation) which can have arbitrary shapes. Also, even if the target structure in two applications are the same, the complexity of the segmentation task can vary significantly from one to another, especially when their underlying imaging modalities and patient populations are different. For example, directly segmenting the left ventricle myocardium from contrast-enhanced MR images (e.g., LGE images) is often more difficult than from MR images without contrast agents, as the anatomical structures are more attenuated by the contrast agent. For cases with certain diseases (e.g., myocardial infarction), the border between the infarcted region and blood pool appears blurry and ambiguous to delineate. As a result, a segmentation network designed for non-contrast enhanced images may not be directly applied to contrast-enhanced images (100). A more sophisticated algorithm is generally required to assist the segmentation procedure. Potential solutions include applying dedicated image pre-processing, enhancing network capacity, adding shape constraints, and integrating specific knowledge about the application.

## 4. Challenges and Future Work

It is evident from the literature that deep learning methods have matched or surpassed the previous state of the art in various cardiac segmentation applications, mainly benefiting from the increased size of public datasets and the emergence of advanced network architectures as well as powerful hardware for computing. Given this rapid process, one may wonder if deep learning methods can be directly deployed to real-world applications to reduce the workload of clinicians. The current literature suggests that there is still a long way to go. In the following paragraphs, we summarize several major challenges in the field of cardiac segmentation and some recently proposed approaches that attempt to address them. These challenges and related works also provide potential research directions for future work in this field.

### 4.1. Scarcity of Labels

One of the biggest challenges for deep learning approaches is the scarcity of annotated data. In this review, we found that the majority of studies uses a fully supervised approach to train their networks, which requires a large number of annotated images. In fact, annotating cardiac images is time consuming and often requires significant amounts of expertise. These methods can be divided into five classes: data augmentation, transfer learning with fine-tuning, weakly and semi-supervised learning, self-supervised learning, and unsupervised learning.

- **Data augmentation**. Data augmentation aims to increase the size and the variety of training images by artificially generating new samples from existing labeled data. Traditionally, this can be achieved by applying a stack of geometric or photometric transformations to existing image-label pairs. These transformations can be affine transformations, adding random noise to the original data, or adjusting image contrast. However, designing an effective pipeline of data augmentation often requires domain knowledge, which may not be easily extendable to different applications. And the diversity of augmented data may still be limited, failing to reflect the spectrum of real-world data distributions. Most recently, several researchers have began to investigate the use of generative models [e.g., GANs, variational AE (219)], reinforcement learning (220), and adversarial example generation (221) to directly learn task-specific augmentation strategies from existing data. In particular, the generative model-based approach has been proven to be effective for one-shot brain segmentation (222) and few-shot cardiac MR image segmentation (223) and it is thus worth exploring for more applications in the future.
- **Transfer learning with fine-tuning**. Transfer learning aims at reusing a model pre-trained on one task as a starting point to train for a second task. The key of transfer learning is to learn features in the first task that are related to the second task such that the network can quickly converge even with limited data. Several researchers have successfully demonstrated the use of transfer learning to improve the model generalization performance for cardiac ventricle segmentation, where they first trained a model on a large dataset and then fine-tuned it on a small dataset (29, 31, 85, 91, 165).
- **Weakly and semi-supervised learning**. Weakly and semi-supervised learning methods aim at improving the learning accuracy by making use of both labeled and unlabeled or weakly-labeled data (e.g., annotations in forms of scribbles or bounding boxes). In this context, several works have been proposed for cardiac ventricle segmentation in MR images. One approach is to estimate full labels on unlabeled or weakly labeled images for further training. For example, Qin et al. (28) and Bai et al. (32) utilized motion information to propagate labels from labeled frames to unlabeled frames in a cardiac cycle whereas (224, 225) applied the expectation maximization (EM) algorithm to predict and refine the estimated labels recursively. Others have explored different approaches to regularize the network when training on unlabeled images, applying multi-task learning (177, 178), or global constraints (226).
- **Self-supervised learning**. Another approach is self-supervised learning which aims at utilizing labels that are *generated automatically* without human intervention. These labels, designed to encode some properties or semantics of the object, can provide strong supervisory signals to pre-train a network before fine-tuning for a given task. A very recent work from Bai et al. (227) has shown the effectiveness of self-supervised learning for cardiac MR image segmentation where the authors used auto-generated anatomical position labels to pre-train a segmentation network. Compared to a network trained from scratch, networks pre-trained on the self-supervised task performed better, especially when the training data was extremely limited.
- **Unsupervised learning**. Unsupervised learning aims at learning without paired labeled data. Compared to the former four classes, there is limited literature about unsupervised learning methods for cardiac image segmentation, perhaps because of the difficulty of the task. An early attempt has been made which applied adversarial training to train a network segmenting LV and RV from CT and MR images without requiring a training set of paired images and labels (121).

In general, transfer learning and self-supervised learning allow the network to be aware of general knowledge shared across different tasks to accelerate learning procedure and to encourage model generalization. On the other hand, data augmentation, weakly and semi-supervised learning allows the network to get more labeled training data in an efficient way. In practice, the two types of methods can be integrated together to improve the model performance. For example, transfer learning can be applied at the model initialization stage whereas data augmentation can be applied at the model fine-tuning stage.

### 4.2. Model Generalization Across Various Imaging Modalities, Scanners, and Pathologies

Another common limitation in DL-based methods is that they still lack generalization capabilities when presented with previously unseen samples (e.g., data from a new scanner, abnormal, and pathological cases that have not been included in the training set). In other words, deep learning models tend to be biased by their respective training datasets. This limitation prevents models to be deployed in the real world and therefore diminishes their impact for improving clinical workflows.

To improve the model performance across MR images acquired from multiple vendors and multiple scanners (53), collected a large multi-vendor, multi-center, heterogeneous labeled training set from patients with cardiovascular diseases. However, this approach may not scale to the real world, as it implies the collection and labeling of a vastly large dataset covering all possible cases. Several researchers have recently started to investigate the use of unsupervised domain adaptation techniques that aim at optimizing the model performance on a target dataset without additional labeling costs. Several works have successfully applied adversarial training to cross-modality segmentation tasks, adapting a cardiac segmentation model learned from MR images to CT images and vice versa (39–41, 228, 229). These type of approaches can also be adopted for semi-supervised learning, where the target domain is a new set of unlabeled data of the same modality (230). Of note, these domain adaptation methods often require the access to unlabeled images in the target domain (e.g., a new scanner, a different hospital), which may not be easy to obtain due to the data privacy and ethics issues. How to collect and share data safely, fairly, and legally across different sites is still an open challenge.

On the other hand, some researchers have started to develop domain generalization algorithms, *without* requiring accessing images from new sites. One stream of works aims to improve the domain generalization ability by extracting domain-independent and robust features or disentangling learned features into domain-specific and domain-invariant components from various seen domains (e.g., multi-center data, multi-modality datasets) to improve the model performance on unseen domains (221, 228, 231). Other researchers have started to adopt data augmentation techniques to simulate various possible data distributions across different domains. For instance, Chen et al. (232) have proposed a data normalization and augmentation pipeline which enables a neural network for cardiac MR image segmentation trained from a single-scanner dataset to generalize well across multi-scanner and multi-site datasets. Zhang et al. (233) applied a similar data augmentation approach to improve the model generalization ability on unseen datasets. Their method has been verified on three tasks including left atrial segmentation from 3D MRI and left ventricle segmentation from 3D ultrasound images.

One bottleneck of augmenting training data for model generalization across different sites is that it is often required to increase the model capacity to compensate for the increased dataset size and variation (232). As a result, training becomes more expensive and challenging. To address this inefficiency problem, active learning (234) has been proposed, which selects the most representative images from a large-scale dataset, reducing labeling workload as well as computational costs. This technique is also related to incremental learning, which aims to improve the model performance by adding new classes incrementally while avoiding a dramatic decrease in overall performance (235). Given the increasing size of the available medical imaging datasets and the practical challenges of collecting, labeling and storing large amounts of images from various sources, it is of great interest to combine domain generalization algorithms with active learning algorithms together to distill a large dataset into a small one but containing the most representative cases for effective and robust learning.

### 4.3. Lack of Model Interpretability

Unlike symbolic artificial intelligence systems, deep learning systems are difficult to interpret and not transparent. Once a network has been trained, it behaves like a “black box,” providing predictions which are not directly interpretable. This issue makes the model unpredictable, intractable for model verification, and ultimately untrustworthy. Recent studies have shown that deep learning-based vision recognition systems can be attacked by images modified with nearly imperceptible perturbations (236–238). These attacks can also happen in medical scenarios, e.g., a DL-based system may make a wrong diagnosis given an image with adversarial noise or even just small rotation, as demonstrated in a very recent paper (239). Although there is no denying that deep learning has become a very powerful tool for image analysis, building resilient algorithms robust to potential attacks remains an unsolved problem. One potential solution, instead of building the resilience into the model, is raising failure awareness of the deployed networks. This can be achieved by providing users with segmentation quality scores (240) or confidence maps, such as uncertainty maps (166) and attention maps (241). These scores or maps can be used as evidence to alert users when failure happens. For example, Sander et al. (166) built a network that is able to simultaneously predict the segmentation mask over cardiac structures and its associated spatial uncertainty map, where the latter one could be used to highlight potential incorrect regions. Such uncertainty information could alert human experts for further justification and refinement in a human-in-the-loop setting.

### 4.4. Future Work

#### 4.4.1. Smart Imaging

We have shown that deep learning-based methods are able to segment images in real-time with good accuracy. However, these algorithms can still fail on those image acquisitions with low image quality or significant artifacts. Although there have been several algorithms developed to avoid this problem by either checking the image quality before follow-up studies (242, 243), or predicting the segmentation quality to detect failures (240, 244, 245), the development of algorithms that can give instant feedback to correct and optimize the image acquisition process is also important despite less explored. Improving the imaging quality can greatly improve the effectiveness of medical imaging as well as the accuracy of imaging-based diagnosis. For radiologists, however, finding the optimal imaging and reconstruction parameters to scan each patient can take a great amount of time. Therefore, a DL-based system that has the potential of efficiently and effectively improving the image quality with less noise is of great need. Some researchers have utilized learning-based methods (mostly are deep learning-based) for better image resolution (62), view planning (246), motion correction (247, 248), artifacts reduction (249), shadow detection (250), and noise reduction (251) after image acquisition. However, combining these algorithms with segmentation algorithms and seamlessly integrating them into an efficient, patient-specific imaging system for high-quality image analysis and diagnosis is still an open challenge. An alternative approach is to directly predict cardiac segmentation maps from undersampled k-space data to accelerate the whole procedure, which bypasses the image reconstruction stage (58).

#### 4.4.2. Data Harmonization

A number of works have reported the existence of missing labels and inconsistent labeling protocols among different cardiac image datasets (27, 232). Variations have been found in defining the end of basal slices as well as the endocardial wall of myocardium (some include papillary muscles as part of the endocardial contours whereas others do not). These inconsistencies can be a major obstacle for transferring, evaluating and deploying deep learning models trained from one domain (e.g., hospital) to another. Therefore, building a standard benchmark dataset like CheXpert (252) that (1) is *large* enough to have substantial data diversity that reflects the spectrum of real-world diversity; (2) has a *standard* labeling protocol approved by experts, is indeed a need. However, directly building such a dataset from scratch is time-consuming and expensive. A more promising way might be developing an automated tool to combine existing public datasets from multiple sources and then to harmonize them to a unified, high-quality dataset. This tool can not only open the door for crowd-sourcing but also enable the rapid deployment of those DL-based segmentation models.

#### 4.4.3. Data Privacy

As deep learning is a data-driven approach, an unavoidable and rife concern is about the data privacy. Regulations, such as The General Data Protection Regulation (GDPR) now play an important role to protect users' privacy and have forced organizations to treat data ownership seriously. On the other hand, from a technical point of view, how to store, query, and process data such that there is no privacy concerns for building deep learning systems has now become an even more difficult but interesting challenge. Building a privacy-preserving algorithm requires to combine cryptography and deep learning together and to mix techniques from a wide range of subjects, such as data analysis, distributed computing, federated learning, differential privacy, in order to achieve models with strong security, fast run time, and great generalizability (253–256). In this respect, Papernot (257) published a report for guidance, which summarized a set of best practices for improving the privacy and security of machine learning systems. Yet, this field is still in its infancy.

## 5. Conclusion

In this review paper, we provided a comprehensive overview of these deep learning techniques used in three common imaging modalities (MRI, CT, ultrasound), covering a wide range of existing deep learning approaches (mostly are CNN-based) that are designed for segmenting different cardiac anatomical structures (e.g., cardiac ventricle, atria, vessel). In particular, we presented and discussed recent progress of deep learning-based segmentation methods in the three modalities, outlined future potential and the remaining limitations of these deep learning-based cardiac segmentation methods that may hinder widespread clinical deployment. We hope that this review can provide an intuitive understanding of those deep learning-based techniques that have made a significant contribution to cardiac image segmentation and also increase the awareness of common challenges in this field that call for future contribution.

## 6. Data Availability Statement

The datasets summarized in Table 6 can be found in their corresponding websites listed below:

- York: http://www.cse.yorku.ca/~mridataset/

- Sunnybrook: http://www.cardiacatlas.org/studies/sunnybrook-cardiac-data/

- LVSC: http://www.cardiacatlas.org/challenges/lv-segmentation-challenge/

- RVSC: http://www.litislab.fr/?projet=1rvsc

- cDEMRIS: https://www.doc.ic.ac.uk/~rkarim/la_lv_framework/fibrosis

- LVIC: https://www.doc.ic.ac.uk/~rkarim/la_lv_framework/lv_infarct

- LASC'13: www.cardiacatlas.org/challenges/left-atrium-segmentation-challenge/

- HVSMR: http://segchd.csail.mit.edu/

- ACDC: https://acdc.creatis.insa-lyon.fr/

- LASC'18: http://atriaseg2018.cardiacatlas.org/data/

- MM-WHS: http://www.sdspeople.fudan.edu.cn/zhuangxiahai/0/mmwhs17/

- CAT08: http://coronary.bigr.nl/centerlines/

- CLS12: http://coronary.bigr.nl/stenoses

- CETUS: https://www.creatis.insa-lyon.fr/Challenge/CETUS

- CAMUS: https://www.creatis.insa-lyon.fr/Challenge/camus.

## Author Contributions

CC, WB, and DR conceived and designed the work. CC, CQ, and HQ searched and read the MR, CT, Ultrasound literature, respectively, and drafted the manuscript together. WB, DR, GT, and JD provided the critical revision with insightful and constructive comments to improve the manuscript. All authors read and approved the manuscript.

### Conflict of Interest

The authors declare that the research was conducted in the absence of any commercial or financial relationships that could be construed as a potential conflict of interest.

## Footnotes

^1^https://www.who.int/cardiovascular_diseases/about_cvd/en/

^2^In a convolution layer *l* with *k*_*l*_ 2D *n* × *n* convolution kernels, each convolution kernel ${\text{CONV}}_{l}^{\left(i\right)},i\in \left(1,k_{l}\right)$ has a weight matrix ${\text{w}}_{l}^{\left(i\right)}$ and a bias term $b_{l}^{\left(i\right)}$ as parameters and can be formulated as: $\text{y}={\text{w}}_{l}^{\left(i\right)}\circ {\text{x}}_{in}+b_{l}^{\left(i\right)}$, where ${\text{w}}_{l}^{\left(i\right)}\in {\mathbb{R}}^{n\times n\times l_{in}},b_{l}^{\left(i\right)}\in \mathbb{R}$, ${\text{x}}_{in}\in {\mathbb{R}}^{H\times W\times l_{in}}$, $\text{y}\in {\mathbb{R}}^{H^{\prime }\times W^{\prime }\times k_{l}}$, *l*_*in*_ denotes the number of channels in the input **x**_*in*_ and ° denotes the convolution operation. Thus, the number of parameters in a convolutional layer is $k_{l}\times \left(n^{2}\times l_{in}+1\right)$. For a convolutional layer with 16 3 × 3 filters where the input is a 28 × 28 × 1 2D gray image, the number of parameters in this layer is 16 × (3^2^ × 1 + 1) = 160. For more technical details about convolutional neural networks, an online tutorial is referred here: http://cs231n.github.io/convolutional-networks.

^3^https://fomoro.com/research/article/receptive-field-calculator

^4^At inference time, the predicted segmentation map for each image is obtained by assigning each pixel with the class of the highest probability: ${\hat{y}}_{i}={\text{argmax}}_{c}\;p_{i}^{c}$.

^5^https://www.nhs.uk/conditions/atrial-fibrillation/

## Abbreviations

**Imaging-related terminology:**
CT: computed tomography
CTA: computed tomography angiography
LAX: long-axis
MPR: multi-planar reformatted
MR: magnetic resonance
MRI: magnetic resonance imaging
LGE: late gadolinium enhancement
RFCA: radio-frequency catheter ablation
SAX: short-axis
2CH: 2-chamber
3CH: 3-chamber
4CH: 4-chamber.
**Cardiac structures and indexes:**
AF: atrial fibrillation
AS: aortic stenosis
AO: aorta
CVD: cardiovascular diseases
CAC: coronary artery calcium
DCM: dilated cardiomyopathy
ED: end-diastole
ES: end-systole
EF: ejection fraction
HCM: hypertrophic cardiomyopathy
LA: left atrium
LV: left ventricle
LVEDV: left ventricular end-diastolic volume
LVESV: left ventricular end-systolic volume
MCP: mixed-calcified plaque
MI: myocardial infarction
Myo: left ventricular myocardium
NCP: non-calcified plaque
PA: pulmonary artery
PV: pulmonary vein
RA: right atrium
RV: right ventricle
RVEDV: right ventricular end-diastolic volume
RVESV: right ventricular end-systolic volume
RVEF: right ventricular ejection fraction
WHS: whole heart segmentation.
**Machine learning terminology:**
AE: autoencoder
ASM: active shape model
BN: batch normalization
CONV: convolution
CNN: convolutional neural network
CRF: conditional random field
DBN: deep belief network
DL: deep learning
DNN: deep neural network
EM: expectation maximization
FCN: fully convolutional neural network
GAN: generative adversarial network
GRU: gated recurrent units
MSE: mean squared error
MSL: marginal space learning
MRF: markov random field
LSTM: Long-short term memory
ReLU: rectified linear unit
RNN: recurrent neural network
ROI: region-of-interest
SMC: sequential monte carlo
SRF: structured random forest
SVM: support vector machine.
**Cardiac image segmentation datasets:**
ACDC: Automated Cardiac Diagnosis Challenge
CETUS: Challenge on Endocardial Three-dimensional Ultrasound Segmentation
MM-WHS: Multi-Modality Whole Heart Segmentation
LASC: Left Atrium Segmentation Challenge
LVSC: Left Ventricle Segmentation Challenge
RVSC: Right Ventricle Segmentation Challenge.
**Others:**
EMBC: The International Engineering in Medicine and Biology Conference
GDPR: The General Data Protection Regulation
GPU: graphic processing unit
FDA: United States Food and Drug Administration
ISBI: The IEEE International Symposium on Biomedical Imaging
MICCAI: International Conference on Medical Image Computing and Computer-assisted Intervention
TPU: tensor processing unit
WHO: World Health Organization.
